# Magnesium bis(monoperoxyphthalate) hexahydrate as mild and efficient oxidant for the synthesis of selenones

**DOI:** 10.3762/bjoc.10.127

**Published:** 2014-06-02

**Authors:** Andrea Temperini, Massimo Curini, Ornelio Rosati, Lucio Minuti

**Affiliations:** 1 Dipartimento di Scienze Farmaceutiche, Università di Perugia, via del Liceo 1, 06123 Perugia, Italy; 2 Dipartimento di Chimica, Biologia e Biotecnologia, Università di Perugia, via Elce di Sotto 8, 06123 Perugia, Italy

**Keywords:** heterocycles, monoperoxyphthalate, oxidation, selenides, selenones

## Abstract

A new, efficient and mild method for the direct oxidation of selenides to selenones using magnesium bis(monoperoxyphthalate) hexahydrate (MMPP) has been developed. Noteworthy this transformation proceeds at room temperature, employs a cheap and safety oxidant and has a broad functional group tolerance. Moreover, the produced selenones could be useful intermediates for the synthesis of different heterocyclic compounds.

## Introduction

**Scheme 1:**
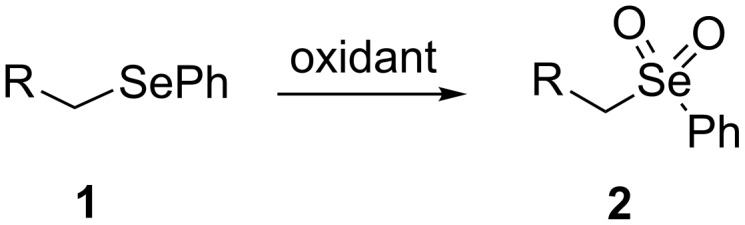
General transformation of selenides to selenones.

Organoselenium compounds have received considerable attention in recent times because they are versatile reagents or intermediates in organic synthesis [1]. Particularly, alkyl phenyl selenones [2–3] represent a valuable compound class due to the ability of the phenylselenonyl group to behave as a good leaving group in substitution reactions (Scheme 1). Thus, the development of efficient and mild methods for their synthesis is of considerable interest.

**Scheme 2:**
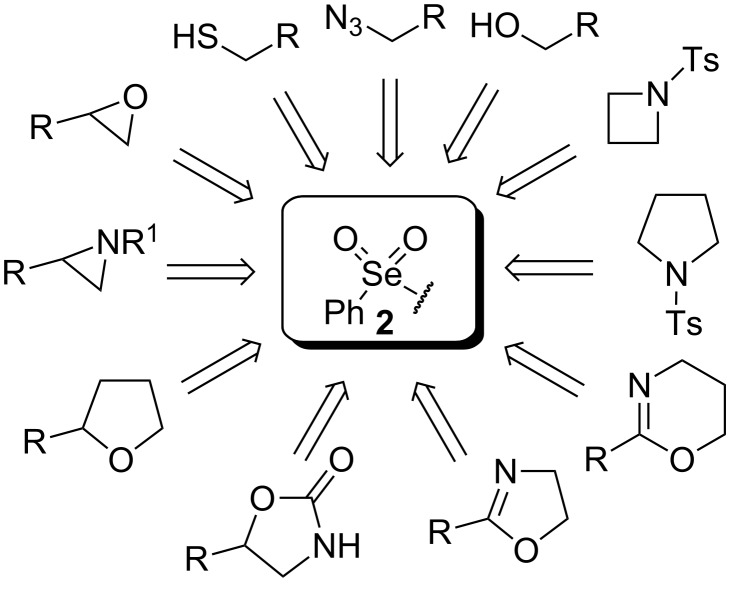
Phenylselenone **2** as useful leaving group for the synthesis of different organic molecules.

A few previous papers report the synthesis of selenones **2** by oxidation of the corresponding selenides **1** with potassium permanganate [4], trifluoroacetic acid [4] and hydrogen peroxide in trifluoroethanol with stoichiometric amounts of phenylseleninic acid [5]. These methods suffer of several disadvantages such as harsh reaction conditions and limited structural features of the substrates; thus, potassium hydrogen persulfate [6] (Oxone) and *m*-chloroperoxybenzoic acid [4,7] (MCPBA) have been employed for the oxidation of different alkyl phenyl selenides to the corresponding selenones. Since the phenylselenonyl group is an excellent leaving group, selenone **2** readily undergoes S_N_2 type substitution reactions with common nucleophiles [7–8] as shown in Scheme 2. A wide range of heterocyclic compounds can be obtained by intramolecular nucleophilic substitution reactions e.g. epoxides from β-hydroxy phenyl selenides [9–10], *N*-benzoyl- and *N***-**tosyl-1,3-oxazolidin-2-ones from β-hydroxyalkyl phenyl selenides [11], *N*-arenesulfonylazetidines from γ-(phenylseleno)alkyl arylsulfonamides [12], *N*-acylaziridines [13], 1,3-oxazolines, dihydrooxazine and pyrrolidines from acylamino phenyl selenides [14]. Interestingly, the intramolecular substitution reaction of a phenylselenonyl group was used on enantioenriched γ-hydroxyalkyl phenyl selenides to obtain optically active 2-substituted tetrahydrofurans [15] (see Scheme 2).

In this article, we investigated the use of magnesium bis(monoperoxyphthalate) hexahydrate (MMPP) as a new oxidant for the straightforward conversion of selenides **1** to selenones **2** under mild experimental conditions. Magnesium bis(monoperoxyphthalate) hexahydrate is a cheap commercially available, relatively stable and easy to use oxidant and it has been employed for the selective oxidation of sulfides to the corresponding sulfoxides, for the preparation of α-methylenecyclohexane [16] and glycosyl sulfoxides [17]. Compared to the widely used peroxy acid MCPBA, MMPP has similar chemical properties, but it is a halogen-free reagent and it is considered safer to use in both small- and large-scale reactions [18]. The only disadvantage of MMPP is its poor solubility in non-polar solvents such as dichloromethane. To the best of our knowledge the use of MMPP for the over oxidation of the selenium atom to the corresponding selenone was not reported before.

## Results and Discussion

Firstly, a solution of selenide **1a** in ethanol was treated with 2.4 equiv of MMPP at room temperature to obtain the corresponding selenone **2a** in 94% yield (Table 1, entry 1). The known oxidation of selenide **1a** with MCPBA in dichloromethane [4] gave the corresponding selenone in 68% yield. It is noteworthy that the reaction byproduct magnesium phthalate is water soluble and easy to separate during work-up; acidification of the water phase and extraction with dichloromethane allows to recover the pure phthalic acid in excellent yield.

**Table 1:** Oxidation of selenides **1a**–**d** to selenones **2a**–**d** with MMPP.

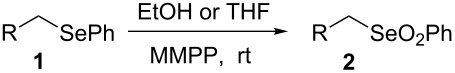					

Entry	Selenide	Solvent	Time (h)	Selenone	Yield (%)

1	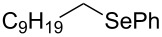 **1a**	EtOH	3	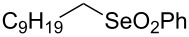 **2a**	94
2	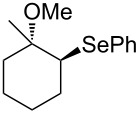 **1b**	THF^a^	3	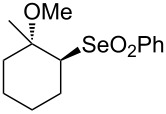 **2b**	54
3	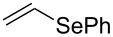 **1c**	THF^a^	2	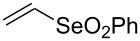 **2c**	80
4	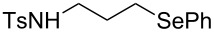 **1d**	EtOH	1	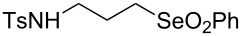 **2d**	63

^a^Dipotassium hydrogen phosphate was added.

This protocol was then extended to the oxidation of selenides **1b**–**d**. The reactions were monitored by simple TLC and good to excellent yields of the selenones **2b**–**d** were obtained after simple work-up without additional purification. Structures of compounds **2a**,**b** and **2d** were assigned by their IR and NMR spectra. The NMR and IR spectra data were in agreement with those of similar compounds reported in the literature [6]. Despite the low solubility of MMPP in tetrahydrofuran, the oxidation of selenides **1b** and **1c** in tetrahydrofuran and in the presence of dipotassium hydrogen phosphate as base, proceeded smoothly and let us to obtain selenones **2b** and **2c** in good yields (Table 1, entry 2 and 3). Selenone **2c** was previously obtained in 60% yield by reaction of **1c** with MCPBA in dichloromethane [19]. Interestingly, selenone **2b** was obtained for the first time by our oxidation procedure performed in tetrahydrofuran as solvent and fully characterized by classical spectroscopic analysis and high resolution mass spectrometry. However, it is important to note that the oxidation of selenide **1b** with MCPBA in methanol did not give selenone **2b** but the corresponding methyl cyclopentyl ketone deriving from ring contraction [20]. On the other hand the oxidation of **1b** with Oxone in methanol and water gave the β-methoxy alcohol through transformation of the C–Se bond into a C–O bond even in the presence of a weak nucleophile like the hydroxy ion [6]. The oxidation of γ-(phenylseleno)alkyl tosylamide **1d** with MMPP (2.4 equiv) in ethanol afforded the corresponding selenone **2d** in 63% yield. Instead, when the same reaction was carried out in tetrahydrofuran, the undesirable olefine derived from the β-elimination of selenoxide intermediate was formed as the main product. Otherwise, when the same reaction was conducted in the presence of powdered potassium hydroxide, selenone intermediate **2d** containing the nucleophilic tosylamide group, readly underwent selective cyclization to give unsubstituted *N*-tosylazetidine (**3d**) in 55% yield (Table 2, entry 1), lower than the yield obtained with MCPBA (100%, [14]). An experiment to replicate the result of Toshimitsu [14] employing MCPBA and potassium hydroxide in ethanol, produced compound **3d** with a comparable yield of 54%. When our procedure was extended to selenides **1e**–**j** containing a nucleophilic nitrogen or oxygen atom, the MMPP oxidation of these selenides, gave the corresponding selenone intermediates which readily undergo intramolecular S_N_2 type substitution reactions to afford a range of heterocyclic compounds (Table 2, entries 2–7) and benzeneseleninic acid. Thus, the oxidation of β-hydroxyalkyl phenyl selenide **1e** gave, in tetrahydrofuran and in the presence of dipotassium hydrogen phosphate, the corresponding oxirane **3e** in 77% yield, resembling the previously reported one by oxidation with MCPBA (73% [9]). The MMPP oxidation of β-benzoylamino phenyl selenide **1f** in methanol afforded exclusively the 1,3-oxazine **3f** in excellent yield (84%).

**Table 2:** Oxidative cyclization with MMPP of functionalized selenides **1e**–**j** to heterocycles **3e**–**j**.

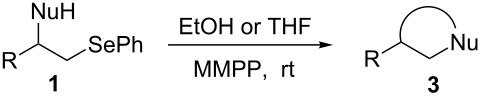					

Entry	Selenide	Solvent	Time (h)	Selenone	Yield (%)

1	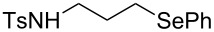 **1d**	EtOH^a^	3	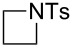 **3d**	55
2	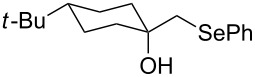 **1e**	THF^b^	16	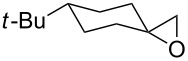 **3e**	77
3	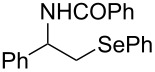 **1f**	MeOH	4	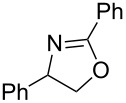 **3f**	84
4	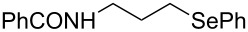 **1g**	MeOH	7	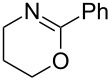 **3g**	67
5	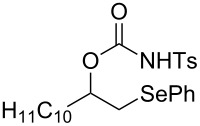 **1h**	THF^b^	10	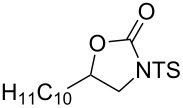 **3h**	94
6	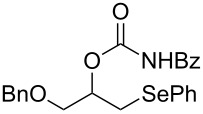 **1i**	THF^c^	6	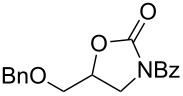 **3i**	82
7	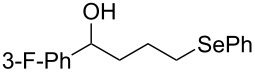 **1j**	MeOH	7	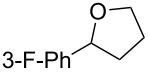 **3j**	69

^a^Potassium hydroxide was added. ^b^Dipotassium hydrogen phosphate was added. ^c^Cyclized in acetone with powdered potassium carbonate.

This result is in accordance with data reported by Toshimitsu [14] for a structural similar compound oxidized with MCPBA. In this case the authors stated that the cyclization by the oxygen atom was very fast and no β-methoxyamide derivative was detected when methanol was employed as the reaction solvent. Similarly the dihydrooxazine **3g** [14] was easily obtained in good yield by the oxidative cyclization of the acylamino phenyl selenide **1g** with MMPP in methanol (Table 2, entry 4) although in lower yield than reported before (90% [14]). As reported in Table 2 (entries 5 and 6), the *N*-tosyl-1,3-oxazolidin-2-one [11] **3h** and the *N*-benzoyl-1,3-oxazolidin-2-one [11] **3i** were easily obtained by MMPP oxidation of the corresponding β-carbamoyloxyalkyl phenyl selenides **1h** and **1i** in tetrahydrofuran. The *N*-substituted-1,3-oxazolidin-2-ones **3h** and **3i** were obtained in 94% and 82% yields respectively as a result of the intramolecular displacement of the phenylselenonyl group by the nitrogen atom of the carbamic group. These results are in accordance with precedent oxidation–cyclization reactions performed with MCPBA (96% and 88% respectively) [11]. The presence of the phenylselenone intermediate in the above reactions was already and unambiguously demonstrated [11]. On the other hand, the oxidation of **1d** with MMPP in ethanol allowed to isolate and to fully characterize (high resolution mass spectra and NMR analysis) the expected selenone intermediate **2d** (Table 1, entry 4). Finally our procedure was applied to the synthesis of 2-substituted tetrahydrofuran **3j** which was previously obtained in 77% yield employing MCPBA in tetrahydrofuran [15]. The oxidation of **1j** with MMPP in tetrahydrofuran proceeded smoothly to give **3j** in poor yield besides the olefine derived from the elimination of the selenoxide intermediate. The use of methanol as the solvent in the oxidative-cyclization reaction of **1j** not only favoured the oxidation reaction, but also suppressed the β-elimination side reaction of the selenoxide intermediate [14]. Thus tetrahydrofuran **3j** was obtained in 69% yield (Table 2, entry 7) as the sole product. It is interesting to note that *N*-heterocyclic compounds **3d** and **3f**–**j** are not obtained when Oxone [6] is employed as oxidant. In these cases the products obtained were the corresponding selenoxides and the β-elimination products.

## Conclusion

In summary, compared to the widely used oxidant MCPBA, MMPP is cheap, stable in the solid state, safer in handling and easier to use. Furthermore, the water soluble reaction byproducts magnesium phthalate and benzeneseleninate anion are generally easy to separate during work-up; the former could be recovered without additional purification as phthalic acid up to 85%, and the latter as diphenyl diselenide [21]. Finally, the oxidation of selenides to selenones described here is compatible with different functional groups, occurs under mild reaction conditions and the yields are comparable with those obtained by means of peroxy acid MCPBA. Application of the reported oxidation methodology to the synthesis of bioactive molecules is currently under investigation.

## Supporting Information

File 1 Experimental procedures, characterization data and copies of ^1^H and ^13^C NMR spectra.
