# Supplementary material for: Magnesium bis(monoperoxyphthalate) hexahydrate as mild and efficient oxidant for the synthesis of selenones
Source: Beilstein J Org Chem. 2014 Jun 2;10:1267–71. doi: 10.3762/bjoc.10.127 (PMC4077369; doi:10.3762/bjoc.10.127)

Supporting Information

for

Magnesium bis(monoperoxyphthalate) hexahydrate as mild and efficient oxidant for the synthesis of selenones

Andrea Temperini*[1], Massimo Curini[1], Ornelio Rosati[1] and Lucio Minuti[2]

Address: [1]Dipartimento di Scienze Farmaceutiche, Università di Perugia, via del Liceo 1, 06123 Perugia, Italy and [2]Dipartimento di Chimica, Biologia e Biotecnologia, Università di Perugia, via Elce di Sotto 8, 06123 Perugia, Italy

Email: Andrea Temperini* - andrea.temperini@unipg.it

* Corresponding author

Dedicated to the memory of Professor Marcello Tiecco.

Experimental procedures, characterization data and copies of ^{1}H and ^{13}C NMR spectra

Table of Contents

Materials and methods S2

Synthesis of Selenone **2a–d** S3

Oxidative-cyclization of Selenides **1d–j** S5

References S8

NMR Spectra S9

Materials and methods. Reactions were conducted in open glassware and were stirred with Teflon-coated magnetic stir bar. Solvents and reagents were used as received unless otherwise noted. Analytical thin layer chromatography (TLC) was performed on silica gel 60 F_{254} pre-coated aluminium foil sheets and visualized by UV irradiation or by use of a $KMnO_4$ stain. Silica gel Kieselgel 60 (70–230 mesh) was used for column chromatography. NMR experiments were obtained at 25 °C on a Bruker DR 200 spectrometer operating at 200 MHz for ^{1}H and 50 MHz for ^{13}C experiments. ^{1}H and ^{13}C chemical shifts (δ) are reported in parts per million (ppm) and they are relative to the residual solvent peak of $CDCl_3$ at δ 7.26 and δ 77.00 in ^{1}H and ^{13}C NMR, respectively. Data are reported as follows: chemical shift (multiplicity, number of hydrogens, coupling constants where applicable, and assignment where possible). Abbreviations are as follows: s (singlet), d (doublet), t (triplet), q (quartet), dd (doublet of doublet), dt (double of triplet), tt (triplet of triplet), m (multiplet), bs (broad signal). Coupling constant (*J*) quoted in Hertz (Hz) to the nearest 0.1 Hz. Infrared (IR) spectra were recorded with a Jasco model 410 spectrometer on a Diffuse Reflectance sampling cell. Only significant absorption maxima (ν_{max}) are reported in wavenumbers (cm^{-1}). HRMS analysis were obtained with a HP–LCMS ESI–qTOF 6200 with Phenomenex Aeris Peptide C18 column (1.7 μm, ID 2.1, 100 mm). Melting points were determined on a Kofler hotstage apparatus and are uncorrected. Compounds **1a** [1], **1b** [2], **1c** [3], **1d** [4], **1g** [4], **1h** [5], **1i** [5] and **1j** [6] were prepared according to the literature. Compound **1e** [7] was synthesized by opening of the corresponding epoxide with phenylselenolate anion [8] whereas compound **1f** [9] from azido selenenylation of styrene.

(Decylselenonyl)benzene (**2a**). To a solution of selenide **1a** (0.25 g, 0.85 mmol) in absolute ethanol (10 mL) at room temperature, magnesium monoperoxyphthalate, MMPP, (1.02 g, 2.04 mmol) was added. The reaction mixture was vigorously stirred until TLC analysis showed that the starting selenide was completely converted into the corresponding selenone (3 h). The mixture was then poured into 10% aqueous sodium carbonate (10 mL) and extracted with ethyl acetate (2 × 20 mL). The organic layer was washed with 10% aqueous sodium carbonate (10 mL), brine (15 mL), dried with sodium sulfate and concentrated in vacuum to afford **2a** as a colorless oil (0.24 g, 94%). FTIR: ν_{max}/cm^{-1} 2924, 1723, 1445, 1286, 926, 879, 744. ^{1}H NMR (200 MHz, $CDCl_3$) δ/ppm 8.01-7.90 (m, 2H, H-Ar), 7.73-7.50 (m, 3H, H-Ar), 3.42 (t, 2H, J = 7.3 Hz, CH_2), 2.05-1.80 (m, 2H, CH_2), 1.48-1.03 (m, 14H, CH_2), 0.82 (t, 3H, J = 6.0 Hz, CH_3). ^{13}C NMR (50 MHz, $CDCl_3$) δ/ppm 139.8, 134.2, 130.2 (2C), 127.0 (2C), 60.0, 31.7, 29.3, 29.1 (2C), 28.8, 28.4, 22.5, 22.3, 14.0. HRMS (ESI-TOF): m/z $[M + H]^+$ calcd for $C_{16}H_{27}O_2Se$ 331.1176; found 331.1168.

At the end of the reaction the oxidant was present as phthalic acid which was extracted by aqueous base. Acidification (pH 2) of the aqueous extract with hydrochloric acid, extraction with dichloromethane (2 x 20 mL) and drying of the organic phase with sodium sulfate produced pure phthalic acid with a 85% yield.

[(*trans*-2-Methoxy-2-methylcyclohexyl)selenonyl]benzene (**2b**). Selenide **1b** (0.28 g, 1.00 mmol) was dissolved in THF (20 mL) and the resulting solution was stirred at room temperature. Powdered dipotassium hydrogen phosphate (0.55 g, 3.00 mmol) and magnesium monoperoxyphthalate (1.20 g, 2.40 mmol) were added and the resulting white suspension was vigorously stirred at room temperature. After 3 h 10% aqueous sodium carbonate (10 mL) was added to the stirred mixture and the solution extracted with ethyl acetate (2 × 20 mL). The organic layer was washed with 10% aqueous sodium carbonate (10 mL), brine (15 mL), dried with sodium sulfate and concentrated in vacuum to produce **2b** as a colorless oil (0.17 g, 54%). FTIR: ν_{max}/cm^{-1} 2940, 1653, 1442, 1119, 965, 861, 744. ^{1}H NMR (200 MHz, $CDCl_3$) δ/ppm 7.75-7.60 (m, 2H, *H*-Ar), 7.58-7.40 (m, 3H, *H*-Ar), 3.31 (s, 3H, OCH_3), 2.81 (dd, 1H, J= 12.0, 5.6 Hz, C*H*), 2.12-2.01 (m, 1H, CH_2), 1.92-1.55 (m, 3H, CH_2), 1.48 (s, 3H, CH_3), 1.39-1.05 (m, 4H, CH_2). ^{13}C NMR (50 MHz,

$CDCl_3$) δ/ppm 140.6, 130.6, 129.3, 129.1, 126.0, 125.8, 76.5, 73.1, 48.7, 37.1, 25.7, 22.7, 20.7, 18.1. HRMS (ESI-TOF): m/z $[M + H]^+$ calcd for $C_{14}H_{21}O_3Se$ 317.0655; found 317.0654.

(Vinylselenonyl)benzene (**2c**). A mixture of selenide **1c** (0.06 g, 0.33 mmol), dipotassium hydrogen phosphate (0.17 g, 0.99 mmol) and magnesium monoperoxyphthalate (0.39 g, 0.70 mmol) in THF (10 mL) was stirred at room temperature for 2 h. The mixture was then poured into 10% aqueous sodium carbonate (10 mL) and extracted with ethyl acetate (2 × 20 mL). The organic layer was washed with 10% aqueous sodium carbonate (10 mL), brine (15 mL), dried with sodium sulfate and concentrated in vacuum to afford **2c** as amorphous light yellow solid (0.06 g, 80%): mp 99-103 °C (from CH_2Cl_2) (lit. [9], 97-102 °C). ^{1}H and ^{13}C NMR spectra are in agreement to that previously reported [9]. ^{1}H NMR (200 MHz, $CDCl_3$) δ/ppm 8.10-7.92 (m, 2H, *H*-Ar), 7.78-7.70 (m, 1H, *H*-Ar), 7.68-7.62 (m, 2H, *H*-Ar), 7.04 (dd, 1H, *J* = 16.6, 9.1 Hz, C*H*), 6.75 (dd, 1H, *J* = 16.6, 2.1 Hz, CH_2), 6.47 (dd, 1H, *J* = 9.1, 2.1 Hz, CH_2); ^{13}C NMR (50 MHz, $CDCl_3$) δ/ppm 141.3, 138.9, 134.2, 131.2, 130.4 (2C), 126.9 (2C).

4-Methyl-*N*-[3-(phenylselenonyl)propyl]benzenesulfonamide (**2d**). To a solution of selenide **1d** (0.11 g, 0.30 mmol) in absolute ethanol (8 mL) at room temperature, magnesium monoperoxyphthalate (0.35 g, 0.71 mmol) was added. The reaction mixture was vigorously stirred until TLC analysis showed that the starting selenide was completely converted into the corresponding selenone (1 h). The mixture was then poured into 10% aqueous sodium carbonate (10 mL) and extracted with ethyl acetate (2 × 20 mL). The organic layer was washed with 10% aqueous sodium carbonate (10 mL), brine (15 mL), dried with sodium sulfate and concentrated in vacuum. The solid residue was washed with ethyl acetate and pure **2d** was obtained as a light yellow solid (0.07 g, 63%); mp 137-139 °C (from EtOAc). FTIR: ν_{max}/cm^{-1} 3164, 1325, 1154, 927, 881, 751. ^{1}H NMR (200 MHz, $CDCl_3$) δ/ppm 8.03 (dd, 2H, *J* = 7.8, 1.4 Hz, *H*-Ar), 7.83-7.64 (m, 5H, *H*-Ar), 7.32 (d, 2H, *J* = 8.0 Hz, *H*-Ar), 5.52 (t, 1H, *J* = 6.5 Hz, N*H*), 3.67 (t, 2H, *J* = 7.0 Hz, CH_2), 3.21 (dt, 2H, *J* = 7.0, 6.5 Hz, CH_2), 2.45 (s, 3H, CH_3), 2.30 (quint, 2H, *J* = 7.0 Hz, CH_2). ^{13}C NMR (50 MHz, $CDCl_3$) δ/ppm 143.6, 141.1, 136.6, 134.5 (2C), 130.4 (2C), 129.8 (2C), 127.0 (3C), 56.9,

41.2, 23.0, 21.5. HRMS (ESI-TOF): m/z $[M + H]^+$ calcd for $C_{16}H_{20}NO_4SSe$ 402.0278; found 402.0274.

Oxidative-Cyclization of 1d–j

1-[(4-Methylphenyl)sulfonyl]azetidine (**3d**). To a solution of phenylseleno amide **1d** (0.20 g, 0.54 mmol) in ethanol (20 mL) at room temperature magnesium monoperoxyphthalate (0.65 g, 1.30 mmol) was added. The reaction mixture was stirred until TLC analysis showed that the starting selenide was completely converted into the corresponding selenone (1 h). Powdered potassium hydroxide (0.24 g, 4.32 mmol) was then added and the consumption of the selenone was monitored by TLC (3 h). The mixture was poured into 10% aqueous sodium carbonate (10 mL) and extracted with ethyl acetate (2 × 20 mL). The organic layer was washed with 10% aqueous sodium carbonate (10 mL), brine (15 mL), dried with sodium sulfate and concentrated in vacuum. Compound **3d** was obtained as amorphous solid (0.06 g, 55%) after chromatography (1: 1 diethyl ether/petroleum ether): mp 118-123 °C (from EtOH). The melting point range, ^{1}H NMR and ^{13}C NMR spectra are in accordance with those obtained from a commercial sample. ^{1}H NMR (200 MHz, $CDCl_3$) δ/ppm 7.78-7.65 (d, 2H, *J* = 7.6 Hz, *H*-Ar), 7.42-7.30 (d, 2H, *J* = 7.6 Hz, *H*-Ar), 3.75 (t, 4H, *J* = 7.65 Hz, CH_2), 2.46 (s, 3H, CH_3), 2.06 (quint, 2H, *J* = 7.6 Hz, CH_2). ^{13}C NMR (50 MHz, $CDCl_3$) δ/ppm 143.8, 131.0, 129.5 (2C), 128.10 (2C), 50.7, 21.3, 15.1.

At the end of the reaction the oxidant was present as phthalate and selenium as benzeneseleninate which were extracted by aqueous base. Acidification (pH 2) of the aqueous extract with hydrochloric acid and extraction with dichloromethane (2 × 20 mL) let us to recover the phthalic acid. The aqueous extract was evaporated and the residue was suspended in MeOH (10 mL). Hydrazine monohydrate (1.5 mmol, 0.07 mL) was added and the suspension stirred until diphenyl diselenide was formed, as indicated by the yellow color. The mixture was then concentrated in vacuo, poured into water and extracted with diethyl ether (3 × 5 mL). The organic layer was dried with sodium sulfate and concentrated in vacuum to afford diphenyl diselenide as a yellow solid (0.06 g, 67%).

***cis*-6-*tert*-Butyl-1-oxaspiro[2.5]octane** (**3e**). To a solution of phenylseleno alcohol **1e** (0.13 g, 0.40 mmol) in THF (10 mL) at room temperature, powdered dipotassium hydrogen phosphate (0.21 g, 1.20 mmol) and magnesium monoperoxyphthalate (0.58 g, 0.96 mmol) were added. The reaction mixture was stirred until TLC analysis showed that the starting selenide was completely converted into the corresponding epoxide (16 h). The mixture was then poured into 10% aqueous sodium carbonate (10 mL) and extracted with ethyl acetate (2 × 20 mL). The organic layer was washed with 10% aqueous sodium carbonate (10 mL), brine (15 mL), dried with sodium sulfate and concentrated in vacuum to afford **3e** as a colorless oil (0.05 g, 77%): ^{1}H NMR spectra is identical to that previously reported [7]. ^{1}H NMR (200 MHz, $CDCl_3$) δ/ppm 2.67 (s, 2H, OC*H*$_2$), 2.02-1.72 (m, 4H, C*H*$_2$), 1.51-1.19 (m, 5H, C*H* and C*H*$_2$), 0.90 (s, 9H, C*H*$_3$). ^{13}C NMR (50 MHz, $CDCl_3$) δ/ppm 58.2, 53.7, 47.1, 33.3 (2C), 32.4, 27.6 (2C), 27.5, 24.8 (2C).

2,4-Diphenyl-4,5-dihydro-1,3-oxazole (**3f**). To a solution of phenylseleno amide **1f** (0.30 g, 0.80 mmol) in methanol (10 mL) at room temperature, powdered magnesium monoperoxyphthalate (0.95 g, 1.92 mmol) was added. The reaction mixture was stirred for 4 h. The mixture was then poured into 10% aqueous sodium carbonate (10 mL) and extracted with ethyl acetate (2 × 20 mL). The organic layer was washed with 10% aqueous sodium carbonate (10 mL), brine (15 mL), dried with sodium sulfate and concentrated in vacuum to afford the 1,3-oxazoline **3f** as a colorless oil (0.15 g, 84%). FTIR: ν_{max}/cm^{-1} 3062, 1723, 1647, 1275, 760, 697 cm^{-1}. ^{1}H NMR (200 MHz, $CDCl_3$) δ/ppm 8.20-7.90 (m, 2H, *H*-Ar), 7.65-7.12 (m, 8H, *H*-Ar), 5.41 (dd, 1H, *J* = 10.1, 8.1 Hz, OC*H*$_2$), 4.82 (dd, 1H, *J* = 10.1, 8.4 Hz, OC*H*$_2$), 4.31 (dd, 1H, *J* = 8.4, 8.1 Hz, NC*H*). ^{13}C NMR (50 MHz, $CDCl_3$) δ/ppm 164.9,142.3, 131.6, 129.1, 129.0, 128.9, 128.8, 128.5, 128.4, 127.8, 127.7, 126.8, 126.6, 74.9, 70.0. HRMS (ESI-TOF): m/z $[M + H]^+$ calcd for $C_{15}H_{14}NO$ ($[M + H]^+$) 224.1075; found 224.1061.

2-Phenyl-5,6-dihydro-4*H*-1,3-oxazine (**3g**). To a solution of phenylseleno amide **1g** (0.25 g, 0.80 mmol) in methanol (15 mL) at room temperature, powdered magnesium monoperoxyphthalate (0.95 g, 1.92 mmol) were added. The reaction mixture was stirred for 7 h. The mixture was then poured into 10% aqueous sodium carbonate (10 mL) and extracted with ethyl acetate (2 × 20 mL).

The organic layer was washed with 10% aqueous sodium carbonate (10 mL), brine (15 mL), dried with sodium sulfate and concentrated in vacuum. The resulting oil was purified by chromatography on SiO_2 (4:6 ethyl ether/petroleum ether) to afford as a colorless oil (0.09 g, 67%): ^{1}H NMR and ^{13}C NMR spectra are agreement to that previously reported [10]. ^{1}H NMR (200 MHz, $CDCl_3$) δ/ppm 7.98-7.80 (m, 2H, *H*-Ar), 7.53-7.26 (m, 3H, *H*-Ar), 4.35 (t, 2H, *J* = 5.5 Hz, OCH_2), 3.63 (t, 2H, *J* = 5.7 Hz, NCH_2), 1.99 (tt, 2H, *J* = 5.7, 5.5 Hz, CH_2). ^{13}C NMR (50 MHz, $CDCl_3$) δ/ppm 155.6, 134.0, 130.3, 128.0 (2C), 126.8 (2C), 65.2, 42.6, 21.9.

5-Decyl-3-[(4-methylphenyl)sulfonyl]-1,3-oxazolidin-2-one (**3h**). To a solution of phenylseleno carbamate **1h** (0.30 g, 0.55 mmol) in THF (20 mL) at room temperature, powdered dipotassium hydrogen phosphate (0.48 g, 1.65 mmol) and magnesium monoperoxyphthalate (0.66 g, 1.33 mmol) were added. The reaction mixture was stirred until TLC analysis showed that the selenone intermediate was completely converted into the cyclized product (10 h). The mixture was then poured into 10% aqueous sodium carbonate (10 mL) and extracted with ethyl acetate (2 × 20 mL). The organic layer was washed with 10% aqueous sodium carbonate (10 mL), brine (15 mL), dried with sodium sulfate and concentrated in vacuum to afford **3h** as a colourless oil (0.22 g, 94%). The ^{1}H NMR and ^{13}C NMR spectra are in agreement to that previously reported [5]. ^{1}H NMR (200 MHz, $CDCl_3$) δ/ppm 7.92 (d, 2H, *J* = 7.6 Hz, *H*-Ar), 7.37 (d, 2H, *J* = 7.6 Hz, *H*-Ar), 4.60-4.40 (m, 1H, C*H*), 4.09 (dd, 1H, *J* = 9.0, 7.9 Hz, CH_2), 3.57 (dd, 1H, *J* = 9.0, 7.2 Hz, CH_2), 2.41 (s, 3H, CH_3), 1.80-1.10 (m, 18H, CH_2), 0.88 (t, 3H, *J* = 6.6 Hz, CH_3). ^{13}C NMR (50 MHz, $CDCl_3$) δ/ppm 151.6, 145.5, 133.8, 129.7 (2 C), 128.0 (2 C), 74.6, 49.5, 34.0, 31.7, 29.3, 29.2, 29.1 (2 C), 28.9, 24.2, 22.5, 21.5, 13.9.

3-Benzoyl-5-[(benzyloxy)methyl]-1,3-oxazolidin-2-one (**3i**). To a solution of phenylseleno carbamate **1i** (0.469 g, 1.00 mmol) in THF (25 mL) at room temperature, powdered dipotassium hydrogen phosphate (0.522 g, 3.00 mmol) and magnesium monoperoxyphthalate (1.19 g, 2.40 mmol) were added. The reaction mixture was stirred until TLC analysis showed that the starting selenide was completely converted into the corresponding selenone (2 h) and then concentrated in vacuum. The residue was suspended in acetone (20 mL) and powdered potassium carbonate was

added (0.69 g, 5.00 mmol). The consumption of the selenone was monitored by TLC (4 h). The mixture was then poured into 10% aqueous sodium carbonate (10 mL) and extracted with ethyl acetate (2 × 20 mL). The organic layer was washed with 10% aqueous sodium carbonate (10 mL), brine (15 mL), dried with sodium sulfate and concentrated in vacuum to afford solid **3i** (0.25 g, 82%): mp 92-94 °C (from CH_2Cl_2) (lit. [8], 93-94 °C). ^{1}H NMR and ^{13}C NMR spectra are identical to that previously reported [8]. ^{1}H NMR (200 MHz, $CDCl_3$) δ/ppm 7.60-7.43 (m, 3H, *H*-Ar), 7.42-7.28 (m, 7H, *H*-Ar), 4.75 (ddt, 1H, *J* = 8.5, 5.8, 3.5 Hz, OC*H*), 4.59 (m, 2H, CH_2), 4.16 (dd, 1H, *J* = 10.8, 8.5 Hz, CH_2), 4.04 (dd, 1H, *J* = 10.8, 5.8 Hz, CH_2), 3.77 (dd, 1H, *J* = 10.8, 3.5 Hz, CH_2), 3.65 (dd, 1H, J = 10.8, 3.5 Hz, CH_2). ^{13}C NMR (50 MHz, $CDCl_3$) δ/ppm 169.4, 152.6, 137.1, 132.7, 131.9 (2 C), 128.6 (3 C), 128.5 (2 C), 127.8, 127.6 (2 C), 73.5, 72.4, 69.7, 45.3.

2-(3-Fluorophenyl)tetrahydrofuran (**3j**); To a solution of phenylseleno alcohol **1j** (0.06 g, 0.02 mmol) in methanol (8 mL) at room temperature, powdered magnesium monoperoxyphthalate (0.25 g, 0.50 mmol) was added. The reaction mixture was stirred for 7 h and then poured into 10% aqueous sodium carbonate (10 mL) and extracted with ethyl acetate (2 × 10 mL). The organic layer was washed with 10% aqueous sodium carbonate (10 mL), brine (15 mL), dried with sodium sulfate and concentrated in vacuum to afford **3j** as a colorless oil (0.02 g, 69%). The ^{1}H NMR and ^{13}C NMR spectra are in agreement to that previously reported [6]. ^{1}H NMR (200 MHz, $CDCl_3$) δ/ppm . 7.30-7.05 (m, 1H, *H*-Ar), 7.05-6.73 (m, 3H, *H*-Ar), 4.90 (t, 1H, *J* = 7.1 Hz, OC*H*), 4.09 (ddd, 1H, *J* = 15.1, 8.2, 1.5 Hz, OCH_2), 3.91 (ddd, 1H, *J* = 15.1, 8.2, 1.5 Hz, OCH_2), 2.38-2.25 (m, 1H, CH_2), 2.05-1.70 (m, 3H, CH_2). ^{13}C NMR (50 MHz, $CDCl_3$) δ/ppm 162.8 (d, J_{CF} = 243.0 Hz), 144.0 (d, J_{CF} = 5.5 Hz), 129.7 (d, J_{CF} = 8.2 Hz), 121.1 (d, J_{CF} = 2.7 Hz) 113.8 (d, J_{CF} = 21.2 Hz), 112.4 (d, J_{CF} = 22.0 Hz), 79.9, 68.7, 34.6, 25.8.

References

[1] Sharpless, K. B.; Young, M. W. *J. Org. Chem.* **1975**, *40*, 947-949.

[2] Toshimitsu, A.; Toshiaki, A; Uemura, S.; Okano, M. *J. Org. Chem.* **1980**, *45*, 1953-1958.

[3] Raucher, S.; Koolpe, G. A. *J. Org. Chem.* **1978**, *43*, 4225-4253.

[4] Toshimitsu, A.; Hirosawa, C.; Tanimoto, S.; Uemura, S. *Tetrahedron Lett*. **1992**, *33*, 4017-4020.

[5] Tiecco, M.; Testaferri, L.; Temperini, A.; Bagnoli, L.; Marini, F.; Santi,C. *Chem. Eur. J.*, **2004**, *10*, 1752-1764.

[6] Minuti,L.; Barattucci, A.; Bonaccorsi, P. M.; Di Gioia, M. L.; Leggio, A.; Siciliano, C.; Temperini, A. *Org. Lett*. **2013**, *15*, 3906-3909.

[7] Corey, E. J.; Chaykovsky, M.; *J. Am. Chem. Soc*. **1965**, *87*, 1353-1364.

[8] Tiecco, M.; Testaferri, L.; Temperini, A.; Bagnoli, L.; Marini, F.; Santi, C. *Synth. Commun*. **1998**, *28*, 2167-2179.

[9] Sternativo, S.; Marini, F.; Dal Verme, F.; Calandriello, A.; Testaferri, L.; Tiecco, M. *Tetrahedron* **2010**, *66*, 6851-6857.

[10] Sinnwell, S.; Ritter, H. *Macromol. Rapid. Commun*. **2006**, *27*, 1335-1340.

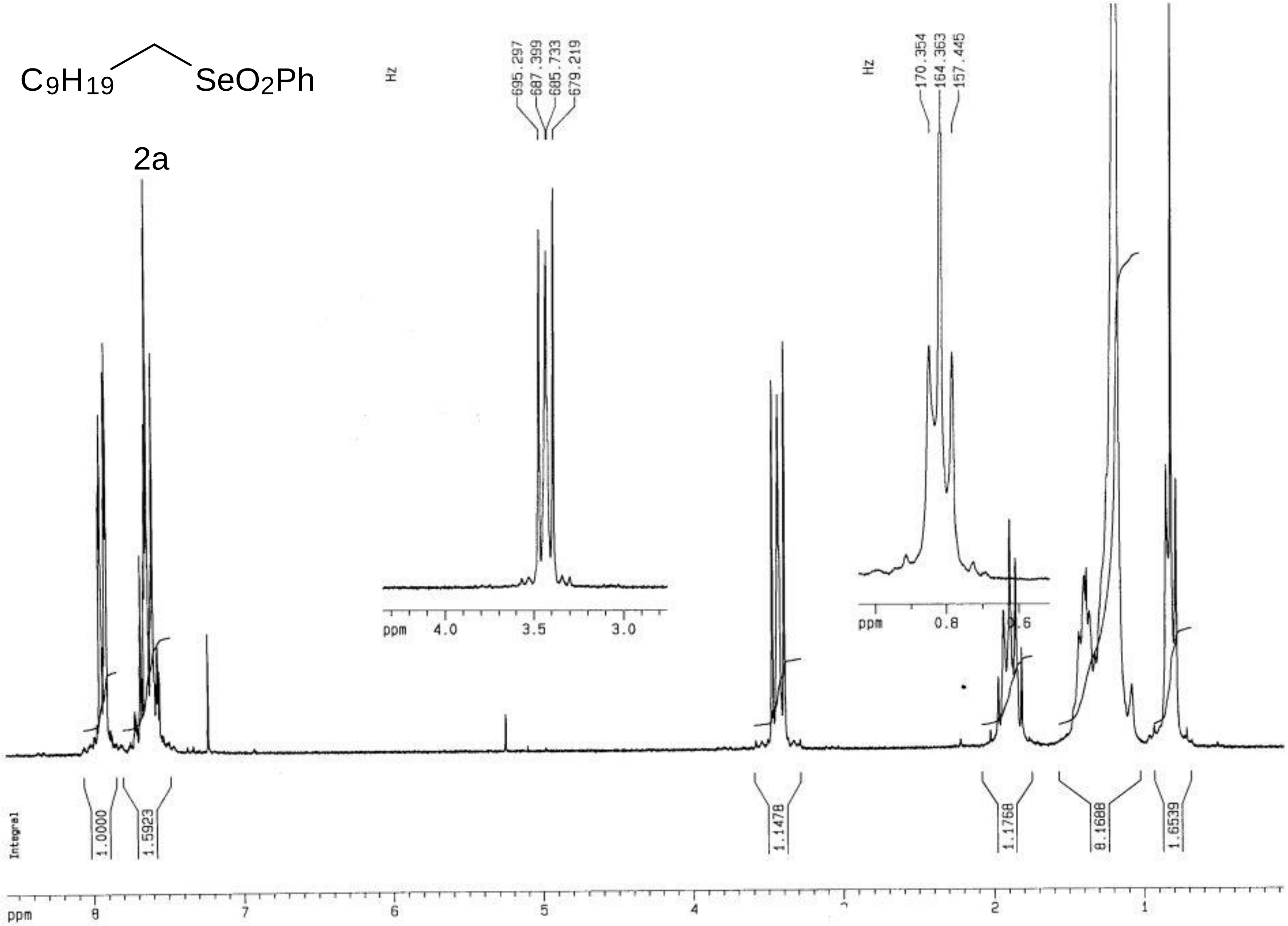

C9H19 SeO2Ph
Hz
695.297
687.399
685.733
679.219
Hz
170.354
164.363
157.445
ppm 4.0 3.5 3.0
ppm 0.8 0.6
Integral
1.0000
1.5923
1.1478
1.1768
8.1688
1.6539
ppm 8 7 6 5 4 2 1

2a

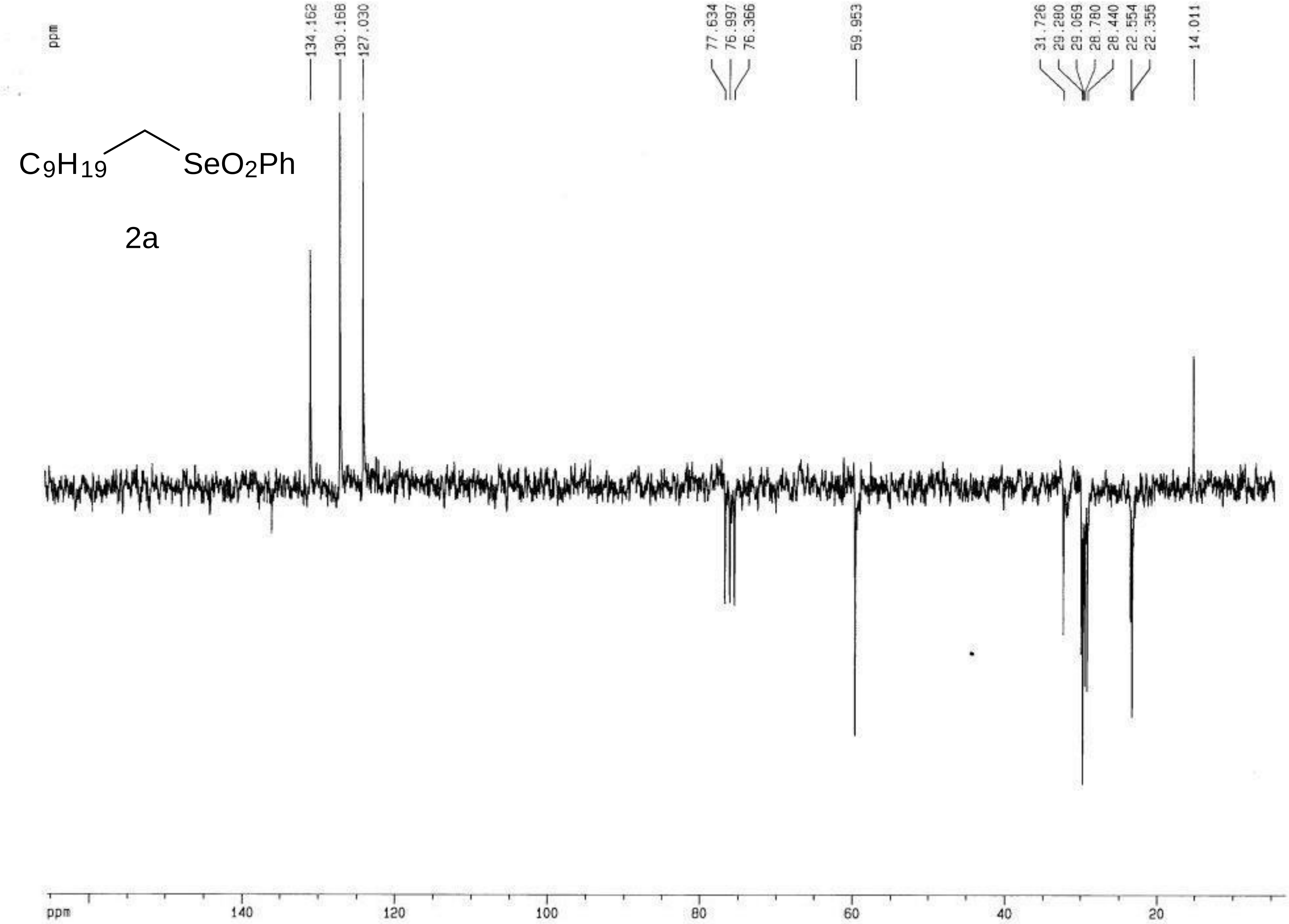
ppm
134.162
130.168
127.030
77.634
76.997
76.366
59.953
31.726
29.280
29.069
28.780
28.440
22.554
22.355
14.011
C_9H_{19}
SeO_2Ph
2a
ppm
140
120
100
80
60
40
20

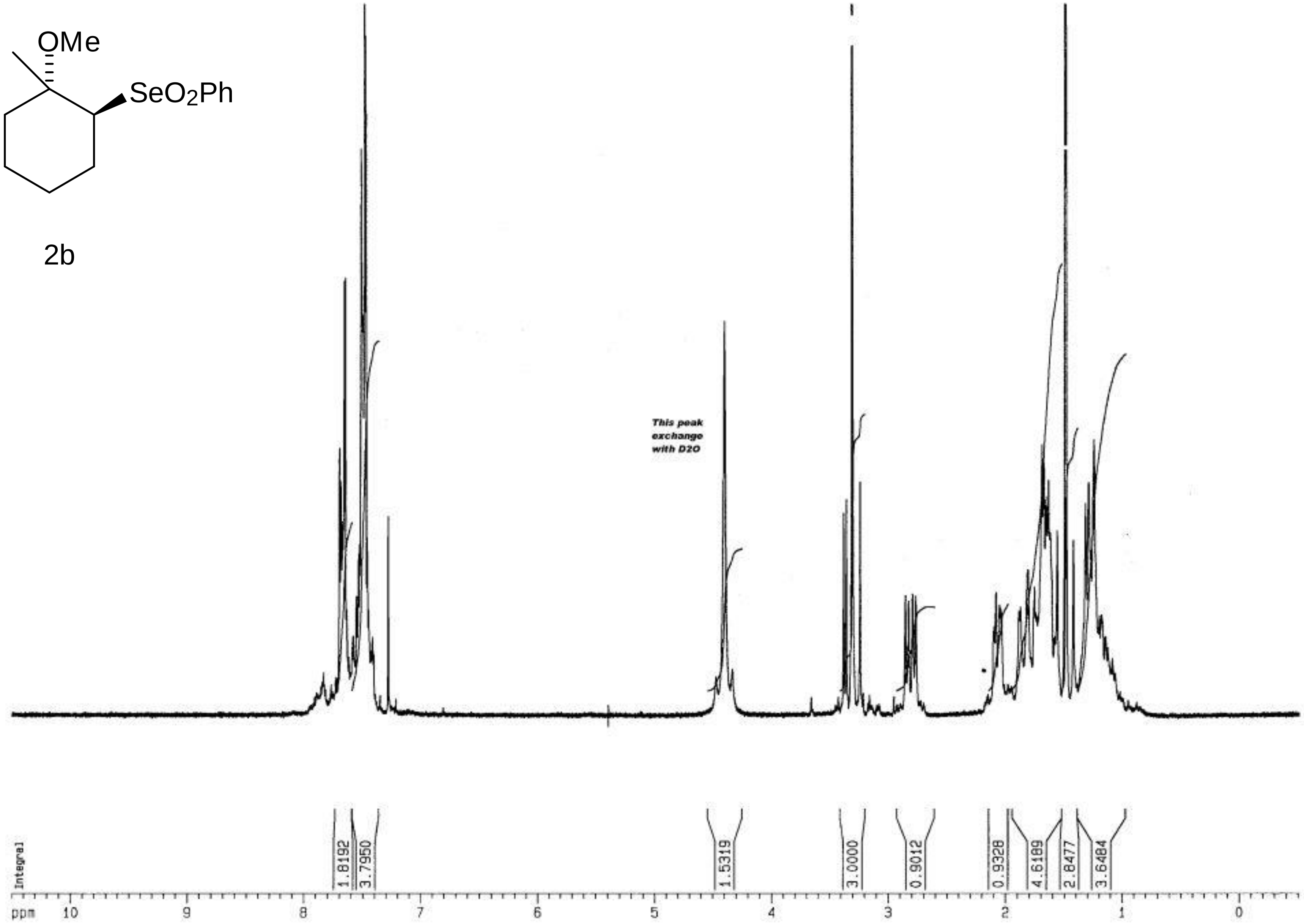
OMe
SeO2Ph

2b

This peak
exchange
with D2O
Integral
1.8192
3.7950
1.5319
3.0000
0.9012
0.9328
4.6189
2.8477
3.6484
ppm
10
9
8
7
6
5
4
3
2
1
0

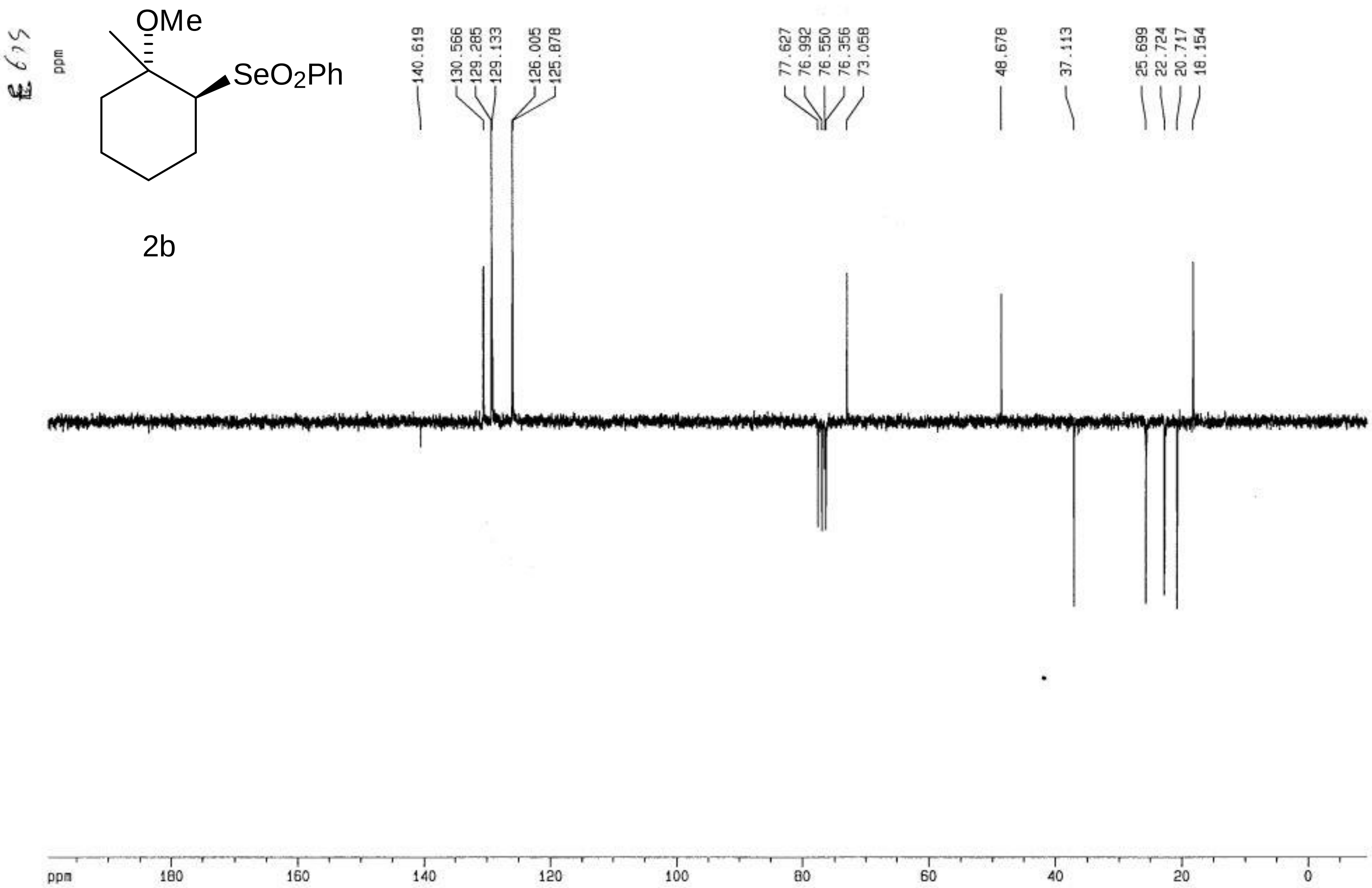
OMe
SeO2Ph
ppm
140.619
130.566
129.285
129.133
126.005
125.878
77.627
76.992
76.550
76.356
73.058
48.678
37.113
25.699
22.724
20.717
18.154
ppm
180
160
140
120
100
80
60
40
20
0

2b

D638

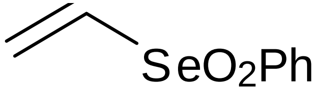
SeO2Ph

2c

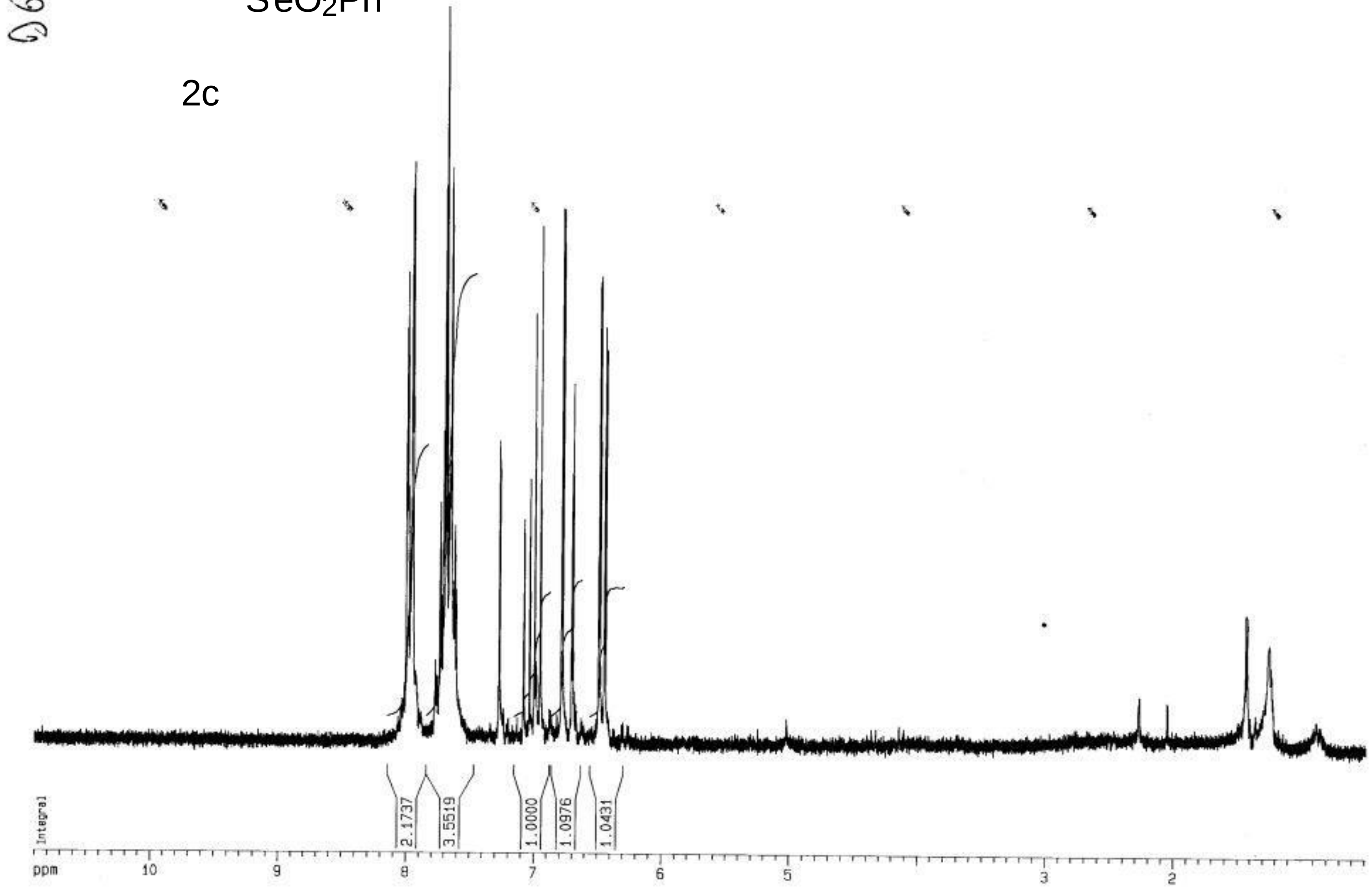
2.1737
3.5519
1.0000
1.0976
1.0431
Integral
ppm
10
9
8
7
6
5
3
2

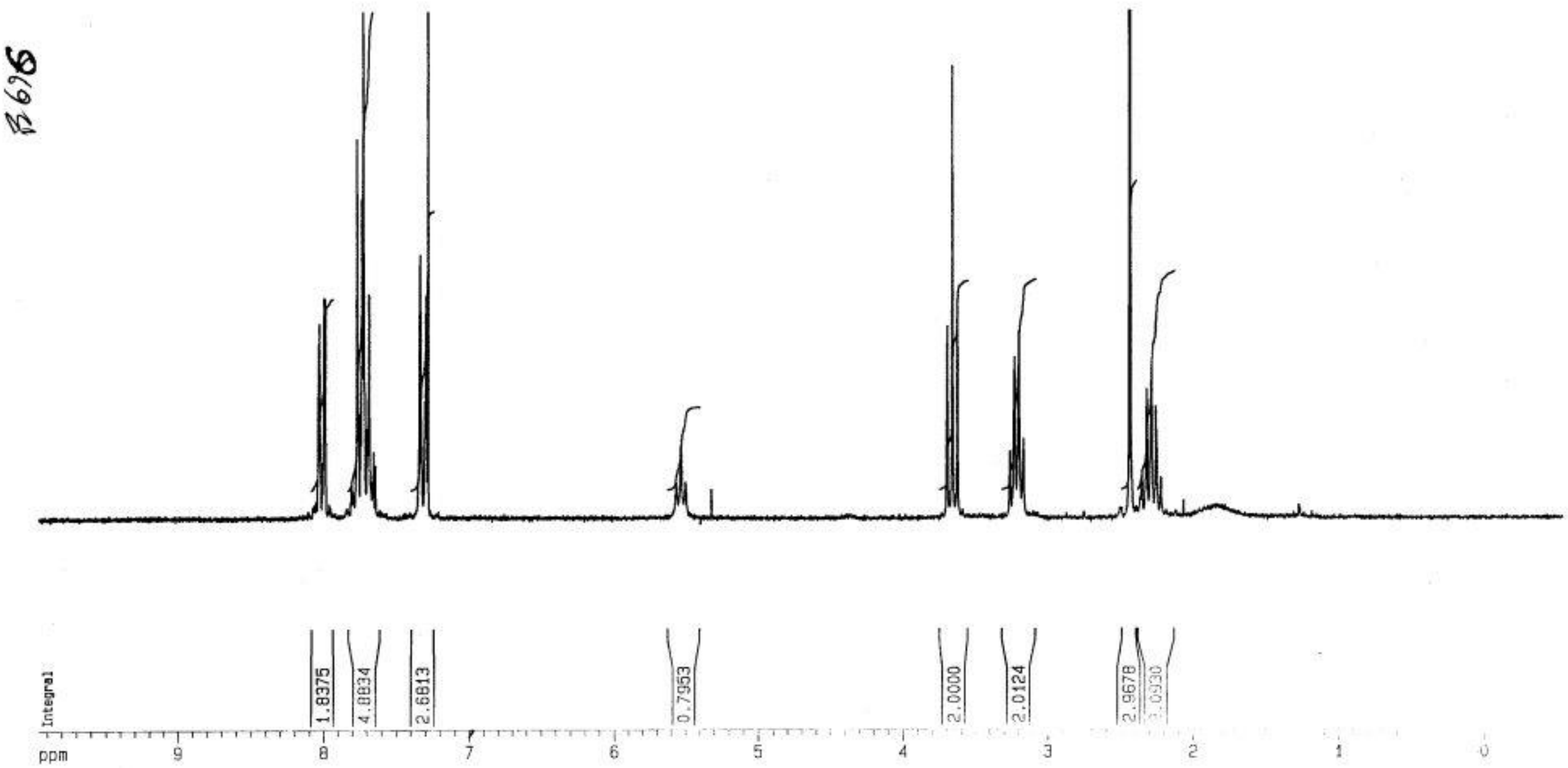
1.8375
4.8834
2.6813
0.7953
2.0000
2.0124
2.9678
3.0930
Integral
ppm
9
8
7
6
5
4
3
2
1
0

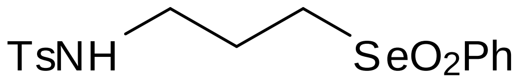
TsNH
SeO2Ph

2d

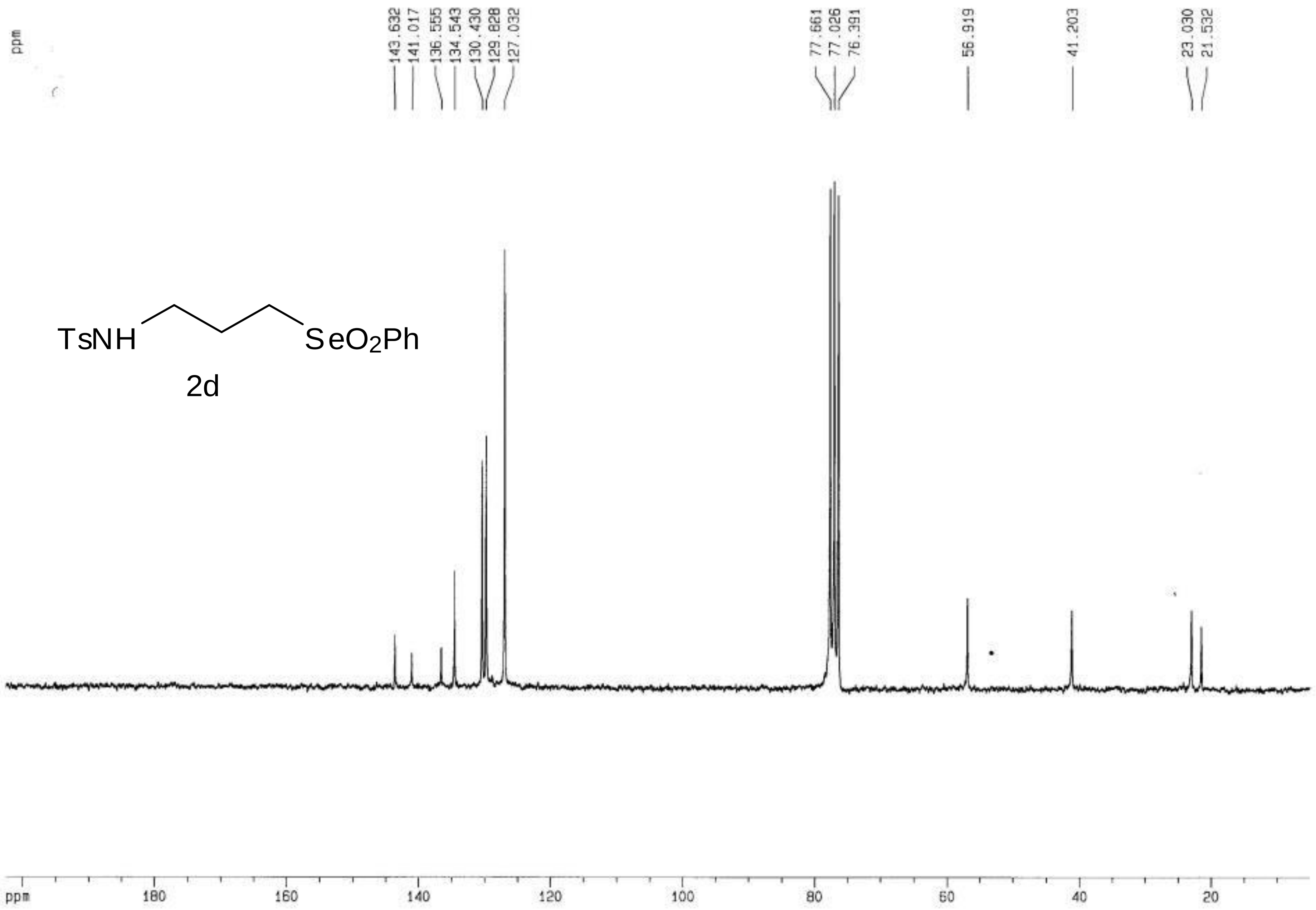

143.632
141.017
136.555
134.543
130.430
129.828
127.032
77.661
77.026
76.391
56.919
41.203
23.030
21.532
TsNH
SeO2Ph
2d
ppm
180
160
140
120
100
80
60
40
20

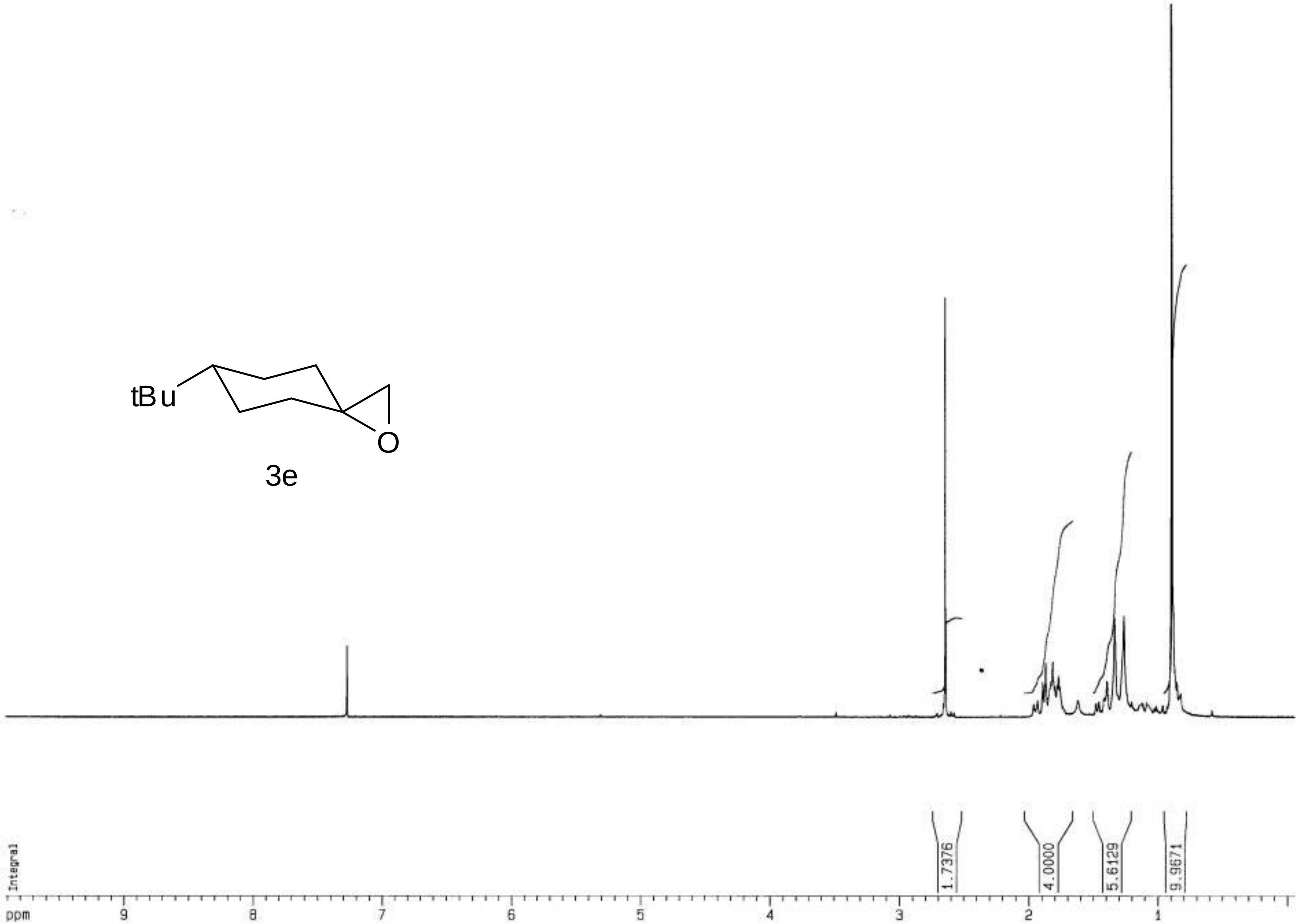

3e

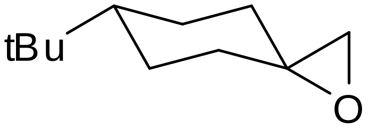
tBu
O

3e

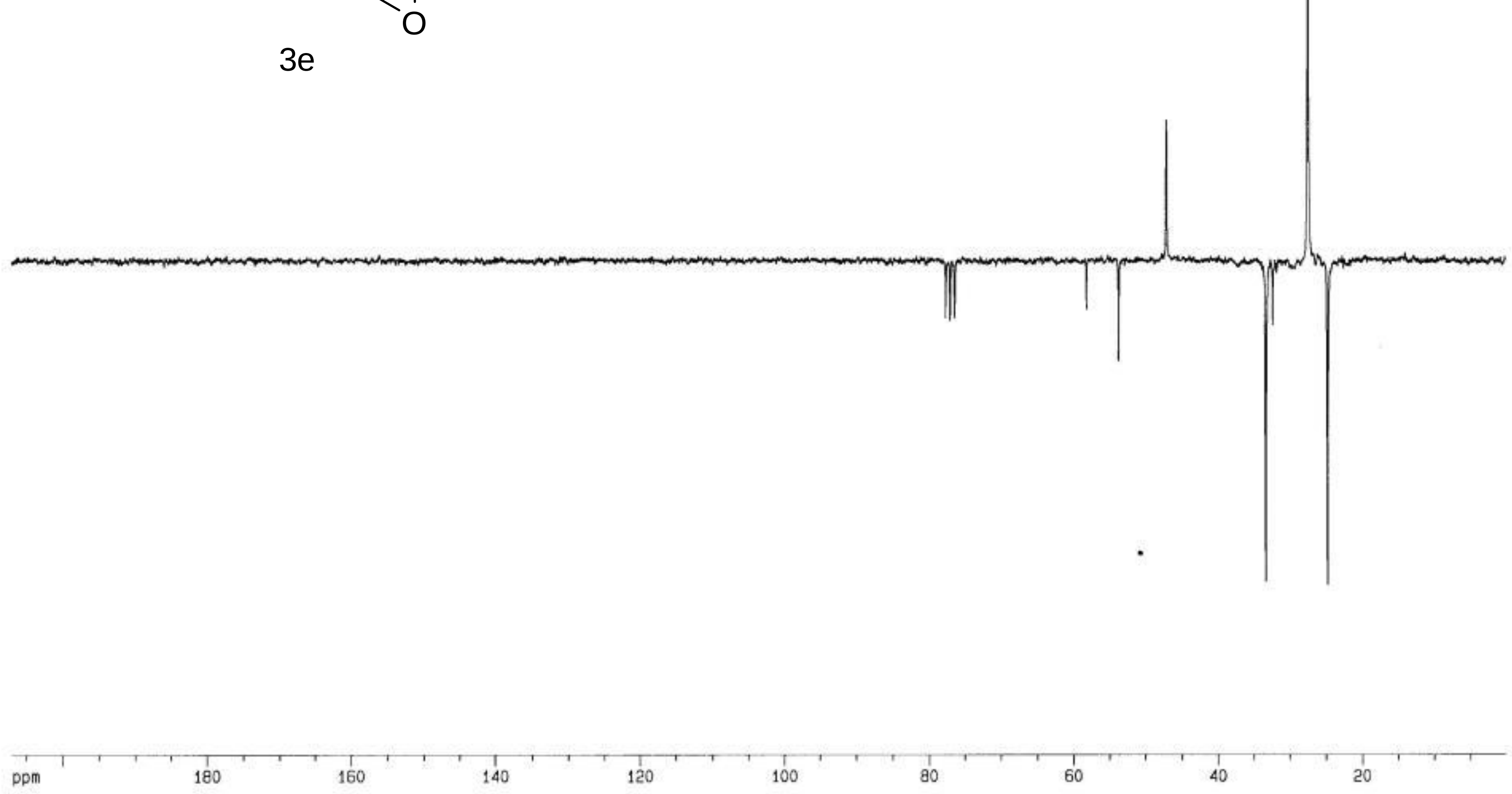
ppm
77.6328
76.9970
76.3600
58.2236
53.6963
47.1061
33.3280
32.4082
27.5589
27.4597
24.7783
ppm
180
160
140
120
100
80
60
40
20

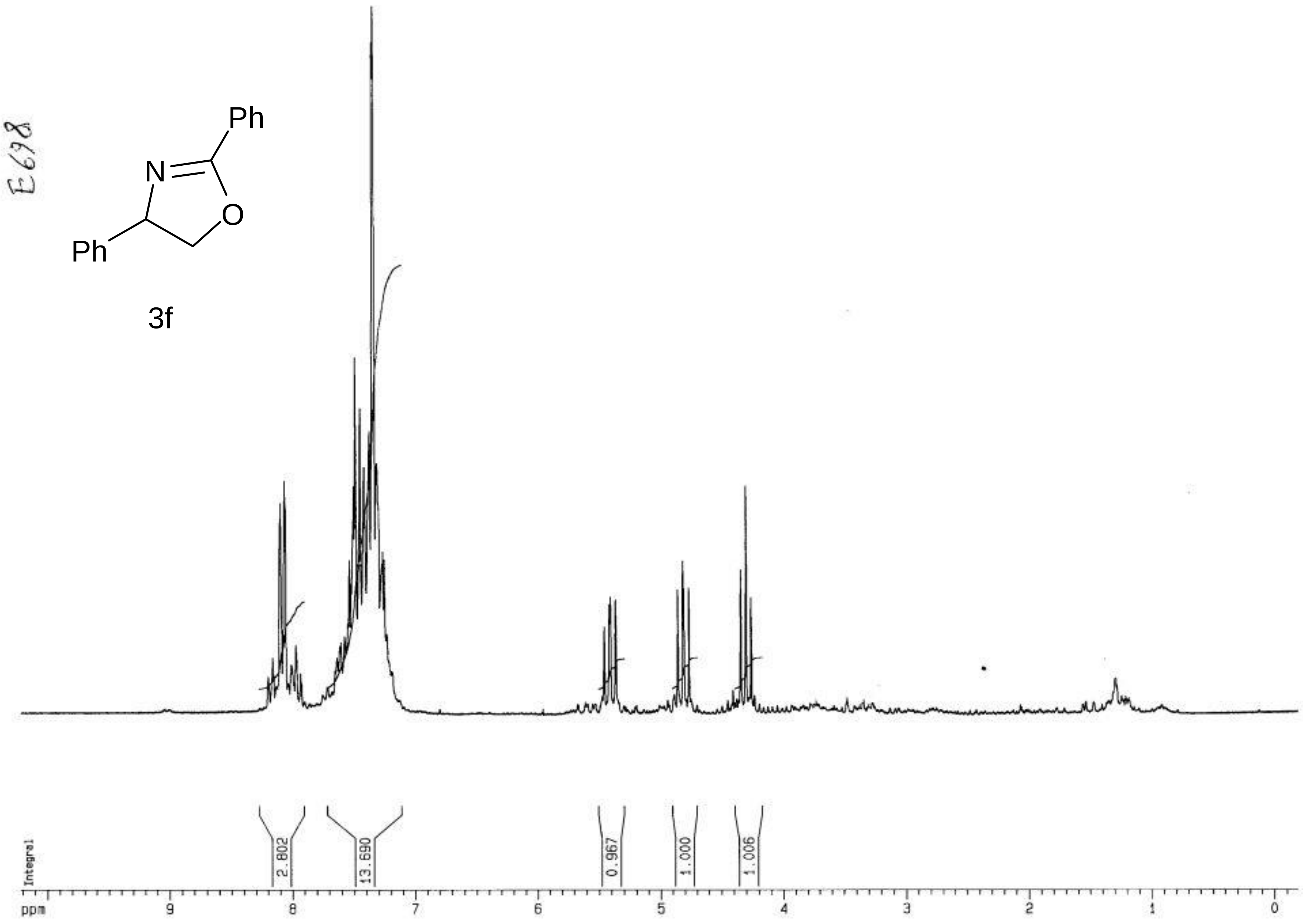
E678
Ph
N
O
Ph
3f
Integral
2.802
13.690
0.967
1.000
1.006
ppm
9
8
7
6
5
4
3
2
1
0

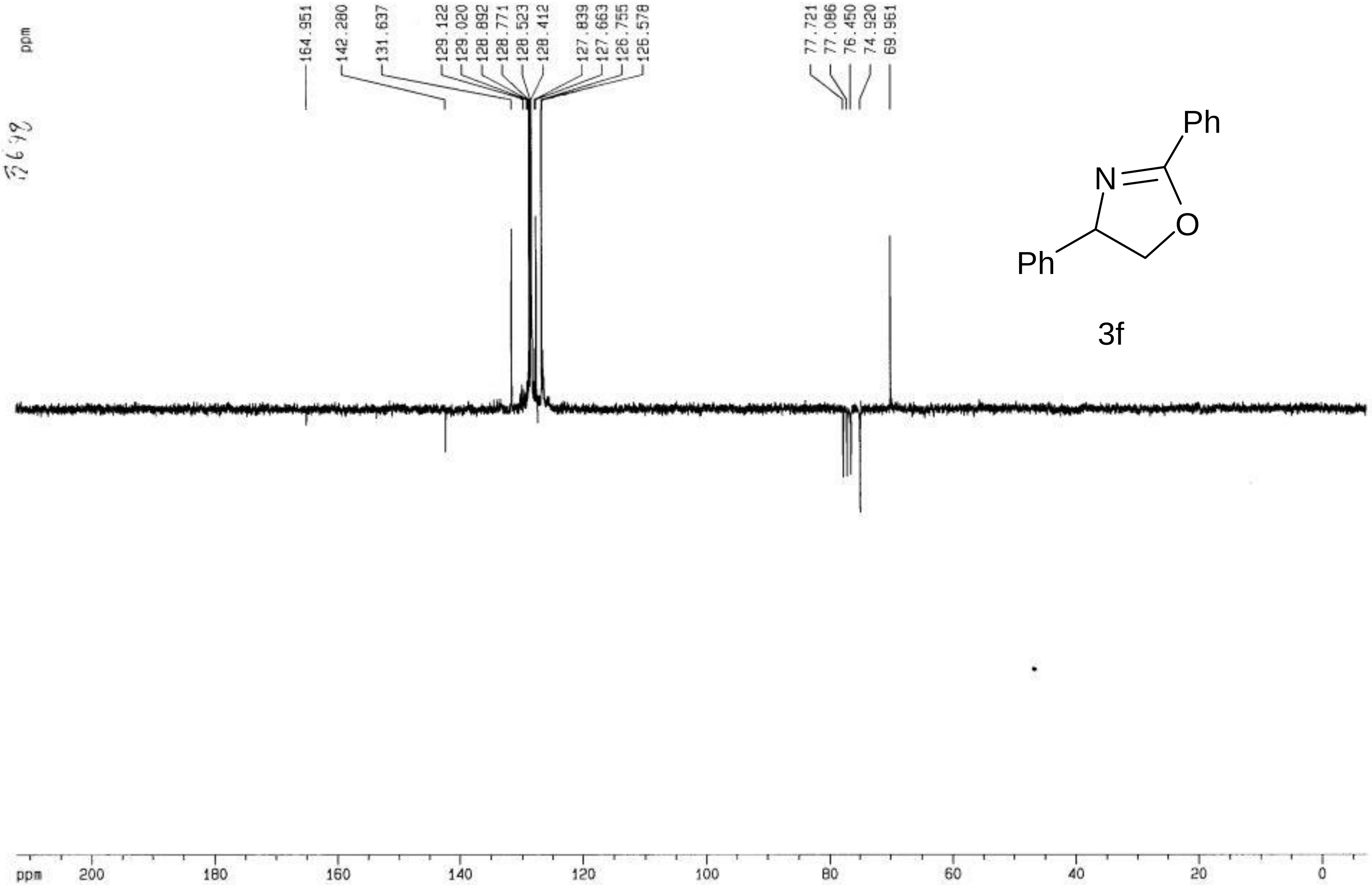
ppm
164.951
142.280
131.637
129.122
129.020
128.892
128.771
128.523
128.412
127.839
127.663
126.755
126.578
77.721
77.086
76.450
74.920
69.961
ppm 200 180 160 140 120 100 80 60 40 20 0
Ph
N
O
Ph

3f

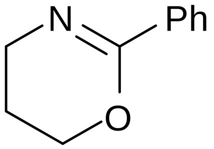

3g

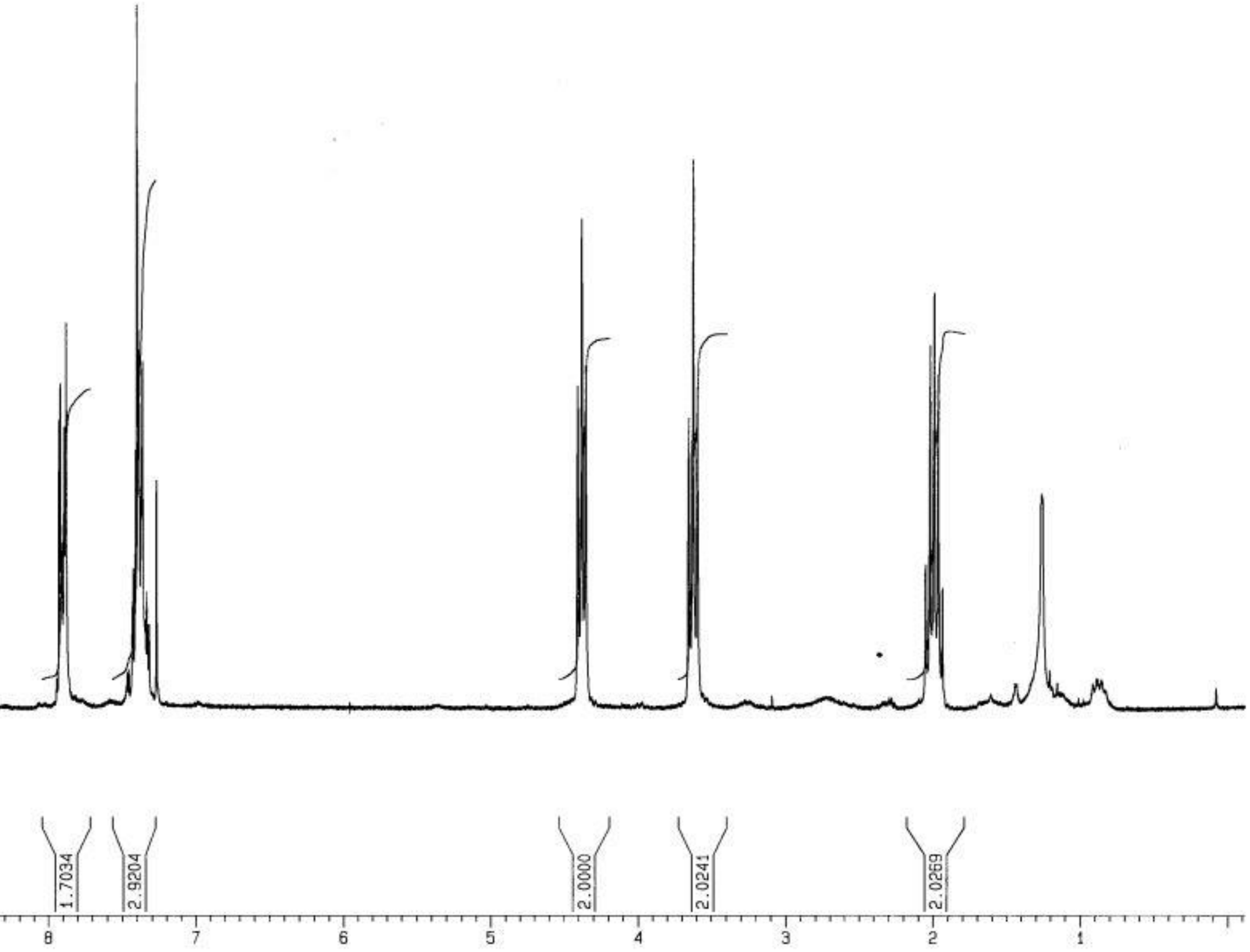

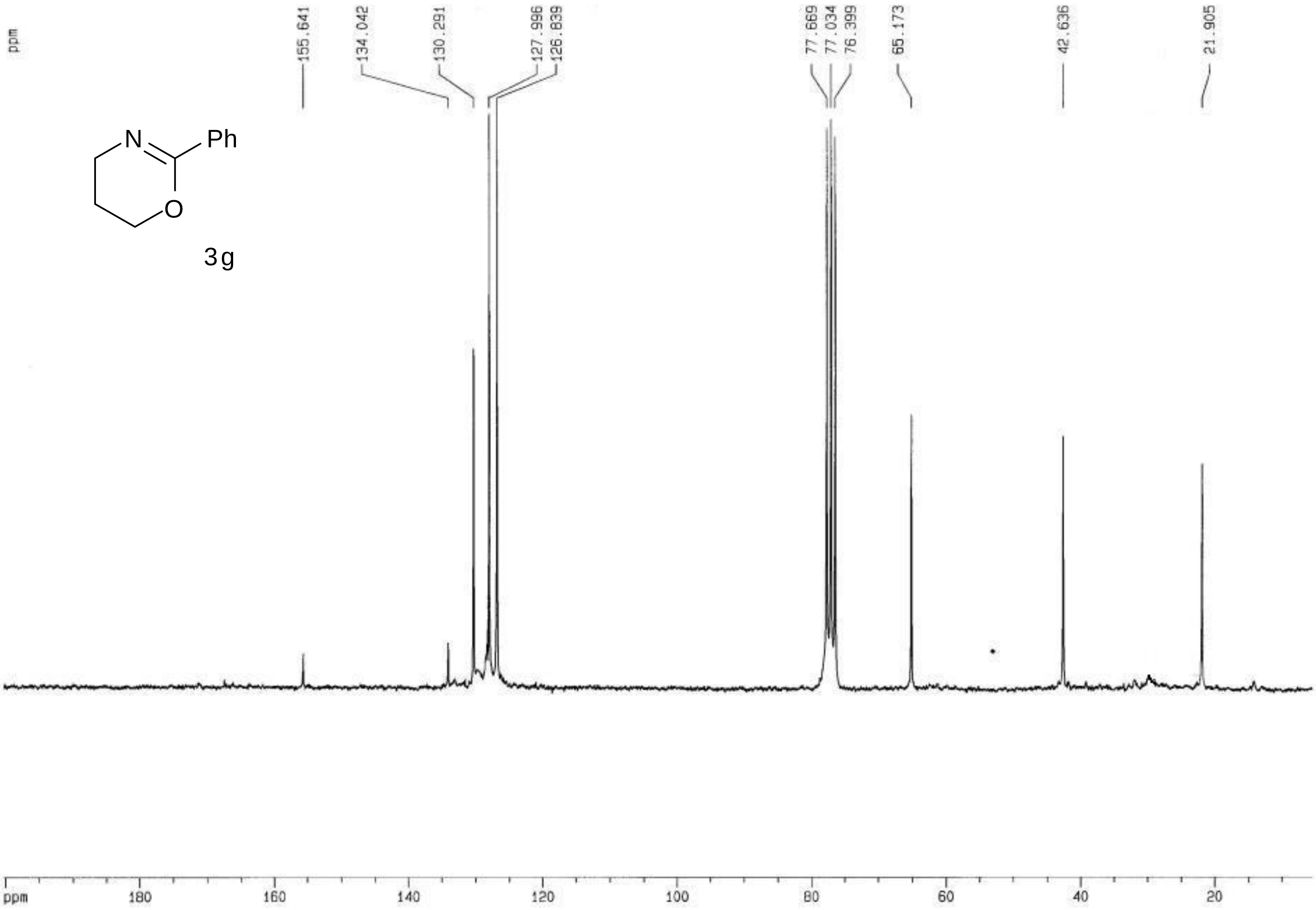
ppm
155.641
134.042
130.291
127.996
126.839
77.669
77.034
76.399
65.173
42.636
21.905
N
Ph
O
3g
ppm
180
160
140
120
100
80
60
40
20

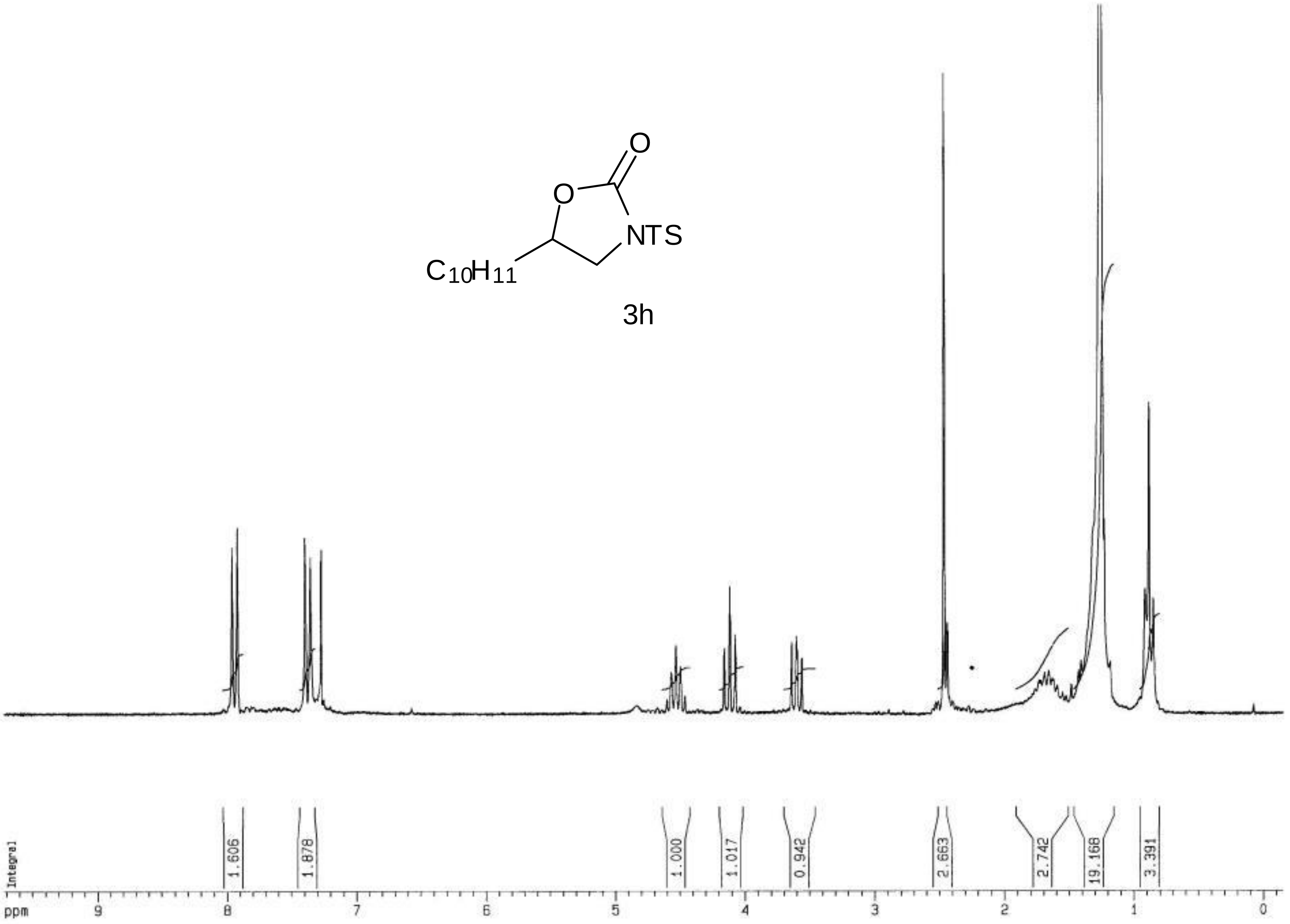
O
O
NTS
C10H11
3h
Integral
1.606
1.878
1.000
1.017
0.942
2.663
2.742
19.168
3.391
ppm
9
8
7
6
5
4
3
2
1
0

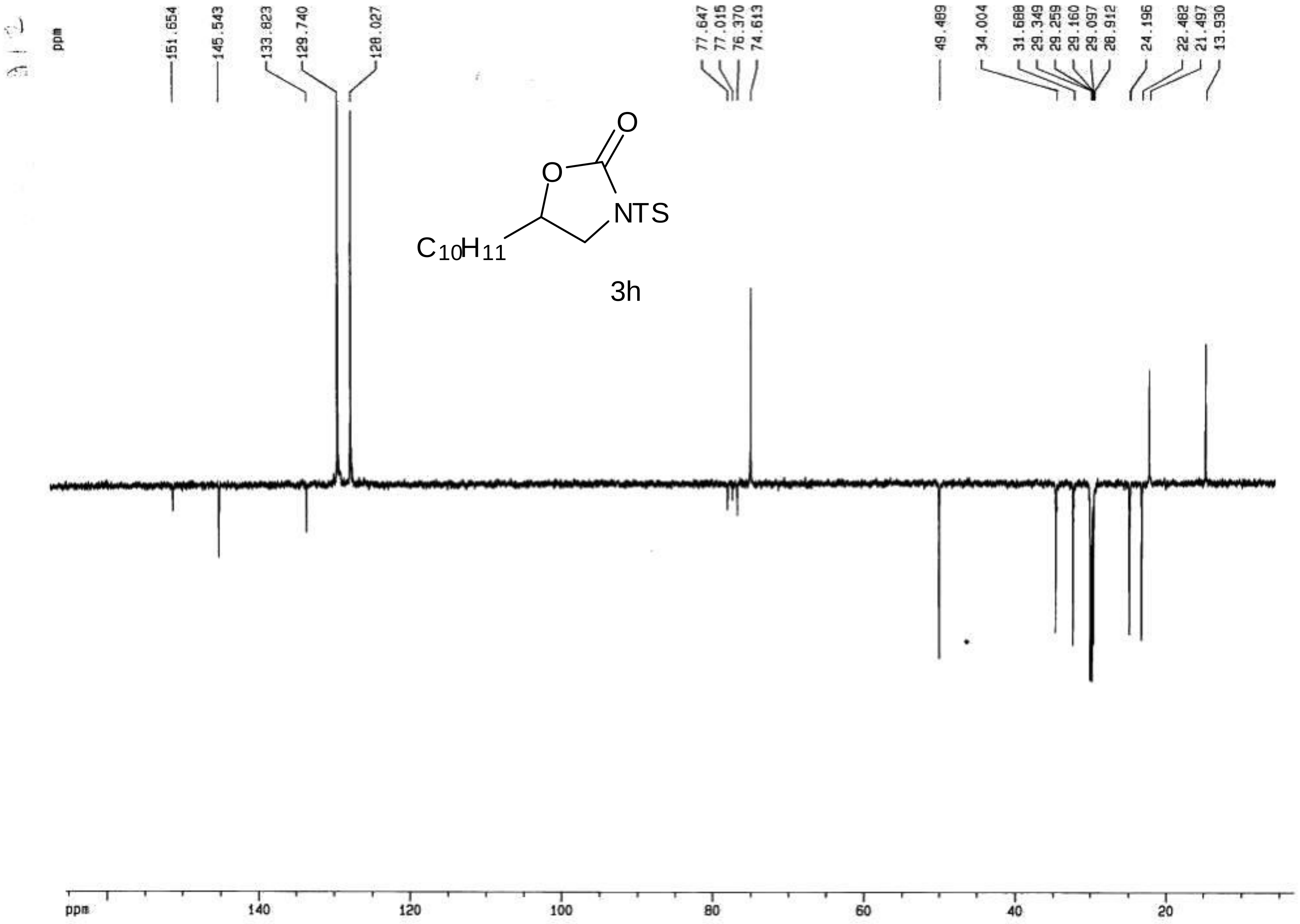
ppm
151.654
145.543
133.823
129.740
128.027
77.647
77.015
76.370
74.613
49.489
34.004
31.688
29.349
29.259
29.160
29.097
28.912
24.196
22.482
21.497
13.930
O
O
NTS
C10H11
3h
ppm
140
120
100
80
60
40
20

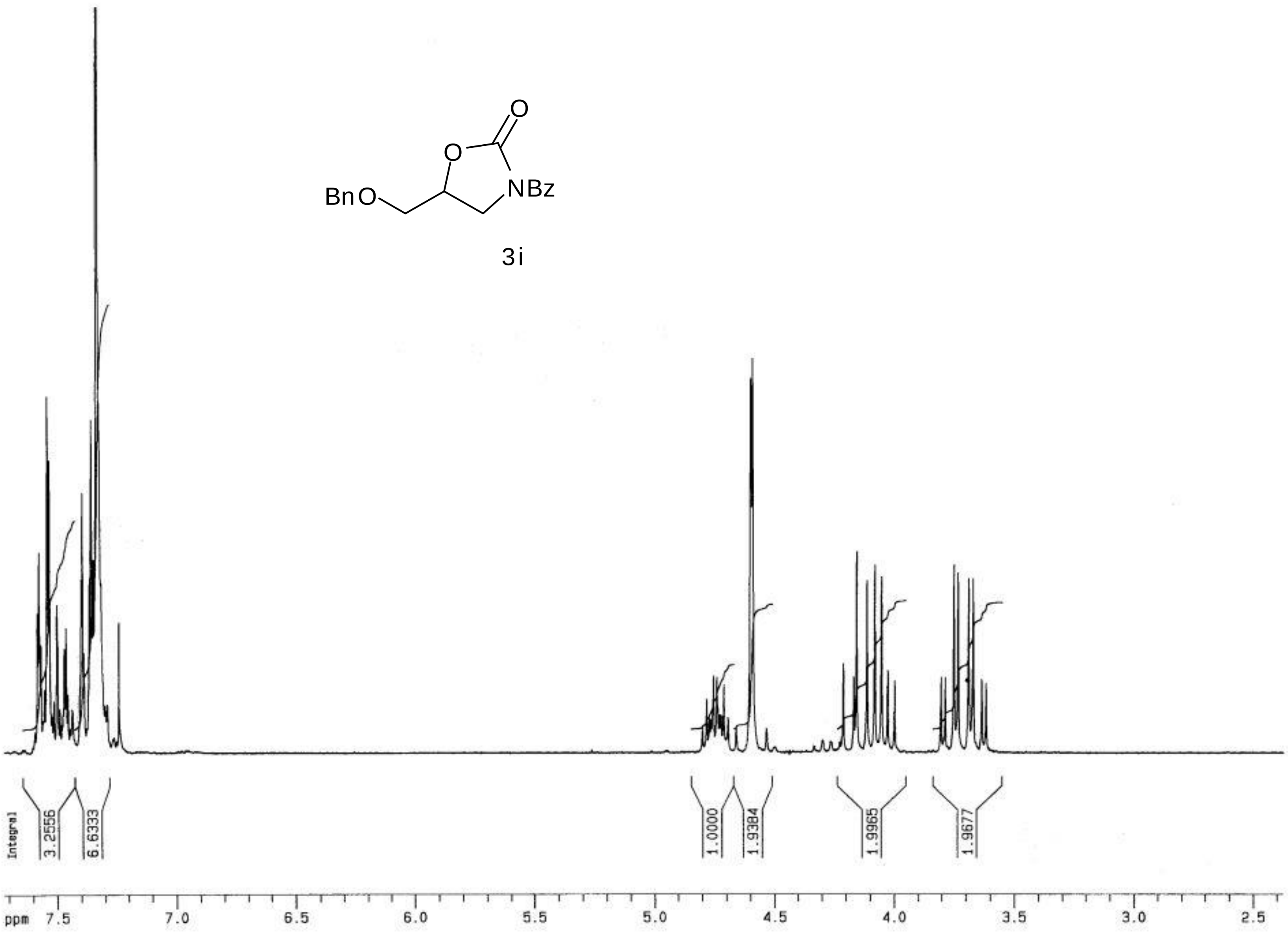

O
O
BnO
NBz
3i
Integral
3.2556
6.6333
1.0000
1.9384
1.9965
1.9677
ppm 7.5 7.0 6.5 6.0 5.5 5.0 4.5 4.0 3.5 3.0 2.5

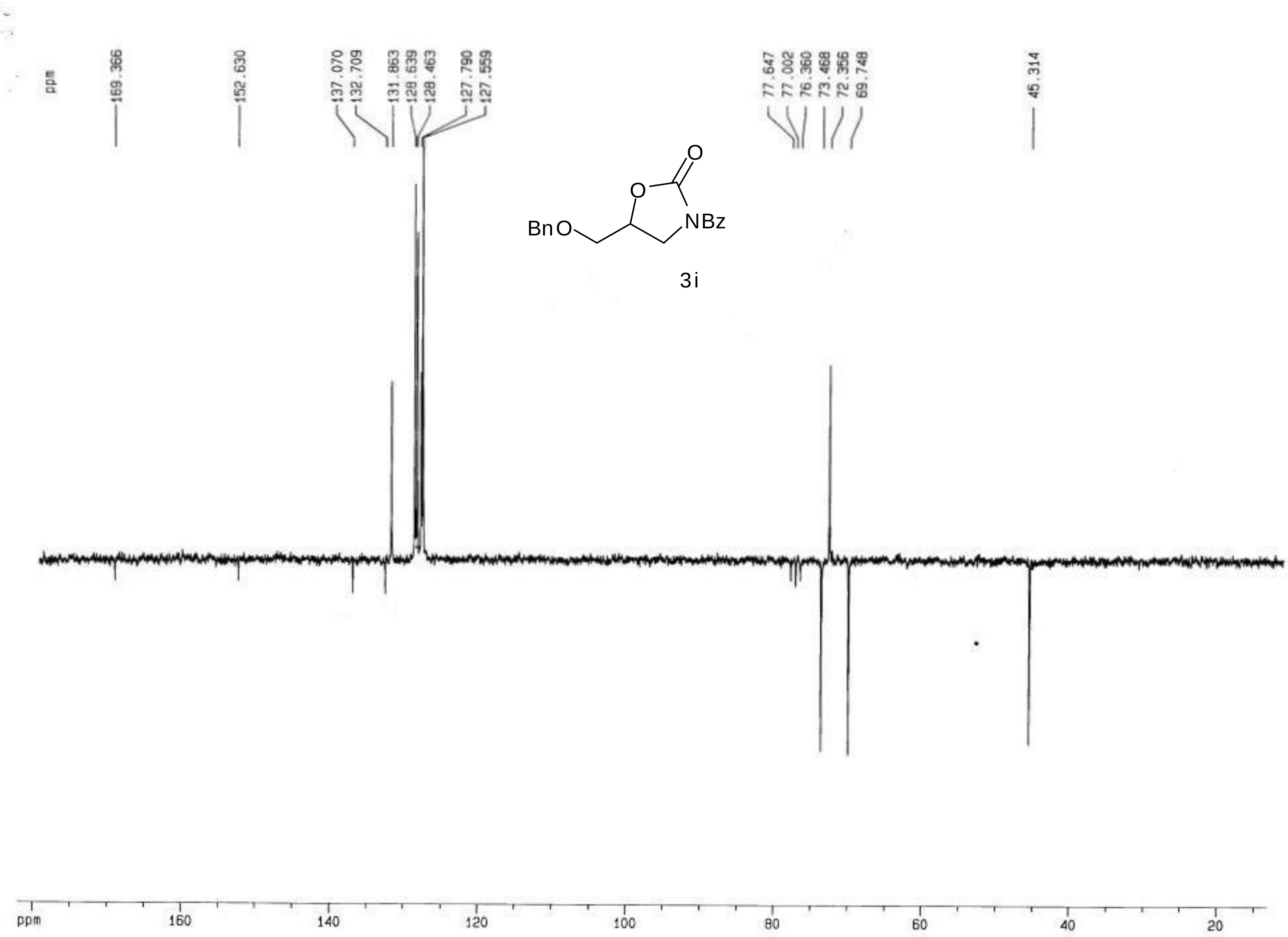

ppm
169.366
152.630
137.070
132.709
131.863
128.639
128.463
127.790
127.559
77.647
77.002
76.360
73.468
72.356
69.748
45.314
O
O
BnO
NBz
3i
ppm
160
140
120
100
80
60
40
20

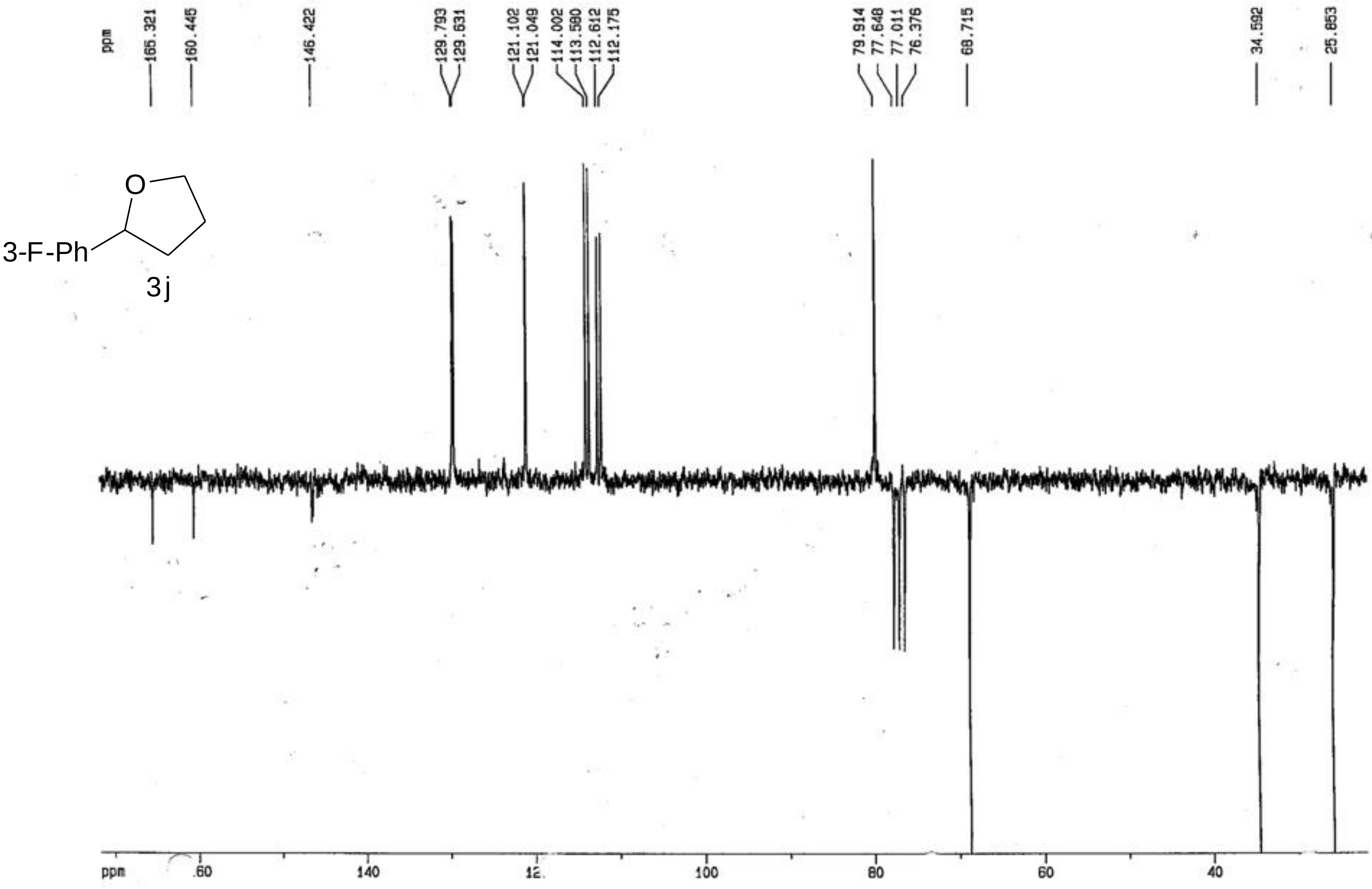
3-F-Ph
O
3j
ppm
165.321
160.445
146.422
129.793
129.631
121.102
121.049
114.002
113.580
112.612
112.175
79.914
77.648
77.011
76.376
68.715
34.592
25.853
ppm
.60
140
12.
100
80
60
40

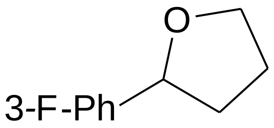

3j

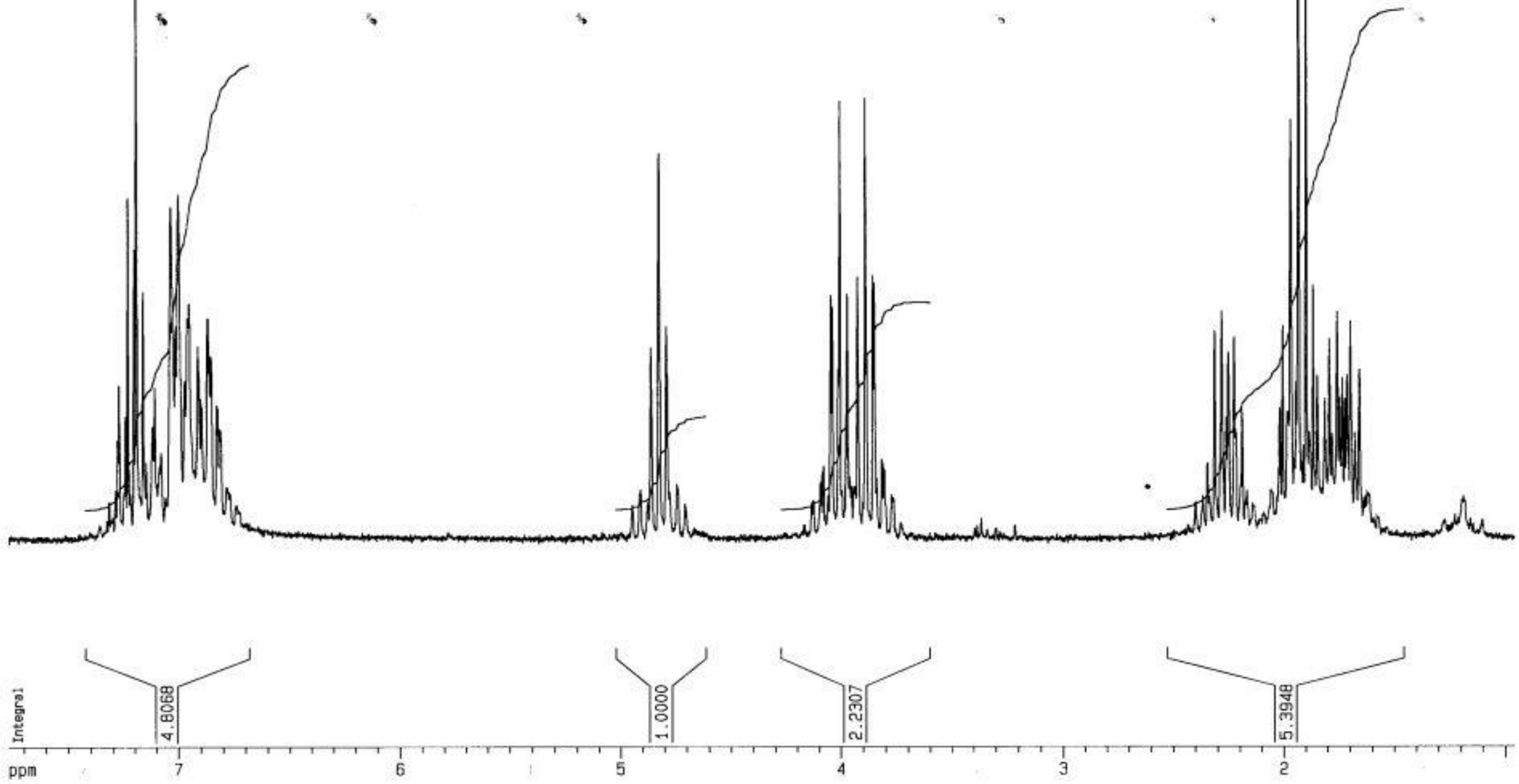

Supplement: File 1 — Experimental procedures, characterization data and copies of 1H and 13C NMR spectra. [file Beilstein_J_Org_Chem-10-1267-s001.pdf]
